# From Metal Stress to Regulated Cell Death: An Evidence Framework for Ferroptosis–Cuproptosis Crosstalk in Cancer

**DOI:** 10.3390/cells15151421

**Published:** 2026-08-05

**Authors:** Andrada-Adelaida Belbe, Lorin-Manuel Pîrlog, Andrei Sporiș, Adela-Diana Pitforodeschi, Alissia-Nicoleta Pilatec, Rareș-Mihai Băilă, Irina Rusu, Mihaela Amelia Dobrescu, Mariela-Sanda Militaru, Irina-Ioana Iordănescu, Andreea Cătană

**Affiliations:** 1 Department of Molecular Sciences, Faculty of Medicine, “Iuliu Hațieganu” University of Medicine and Pharmacy, 400012 Cluj-Napoca, Romania; adelaidapatrascanu@gmail.com (A.-A.B.); adela.diana.p1@gmail.com (A.-D.P.); pilatecalissia@gmail.com (A.-N.P.); raresbaila@gmail.com (R.-M.B.); irinarusu2003@gmail.com (I.R.); sanda.militaru@umfcluj.ro (M.-S.M.); catanaandreea@elearn.umfcluj.ro (A.C.); 2 Department of Medical Genetics, Clinical Emergency Hospital for Children, 400370 Cluj-Napoca, Romania; sporisandrei99@gmail.com; 3 Department of Medical Genetics, University of Medicine and Pharmacy of Craiova, 2, Petru Rareș St., 200349 Craiova, Romania; amelia.dobrescu@umfcv.ro; 4 Regional Centre of Medical Genetics Dolj, Emergency County Hospital Craiova, 200642 Craiova, Romania; 5 Regional Laboratory Cluj-Napoca, Department of Medical Genetics, Regina Maria Health Private Network, 400363 Cluj-Napoca, Romania; 6 Genetic Centre Laboratory, Department of Medical Genetics, Regina Maria Health Private Network, 011376 Bucharest, Romania; irina.iordanescu@reginamaria.ro; 7 Genetics Department, “Carol Davila” University of Medicine and Pharmacy, 020027 Bucharest, Romania; 8 Department of Oncogenetics, “Prof. Dr. I. Chiricuță” Institute of Oncology, 400015 Cluj-Napoca, Romania

**Keywords:** ferroptosis, cuproptosis, cancer metabolism, regulated cell death, lncRNA, metal homeostasis, *GPX4*, FDX1, lipid peroxidation, protein lipoylation

## Abstract

**Highlights:**

**What are the main findings?**

- Ferroptosis and cuproptosis are distinct but connected metal-dependent forms of regulated cell death that expose cancer-specific metabolic liabilities.
- The manuscript separates validated pathway regulators from correlative genes, lncRNAs, prognostic signatures, and generic oxidative injury markers.

**What are the implications of the main findings?**

- Mechanism-guided translation requires integrated biomarkers of metal handling, lipid remodelling, antioxidant reserve, mitochondrial metabolism, and protein lipoylation.
- Therapeutic strategies should prove pathway engagement with pharmacodynamic endpoints rather than relying on ROS accumulation or reduced viability alone.

**Abstract:**

Resistance to apoptosis, metabolic plasticity, and redox adaptation are major contributors to cancer progression and treatment failure. Ferroptosis and cuproptosis have therefore emerged as metal-dependent forms of regulated cell-death programs with potential relevance for tumours that survive conventional therapy. Ferroptosis is driven by iron-dependent phospholipid peroxidation when glutathione peroxidase 4 (GPX4)-dependent and parallel antioxidant systems fail, whereas cuproptosis depends on mitochondrial copper engagement of lipoylated tricarboxylic-acid-cycle proteins, lipoylated-protein aggregation, iron–sulfur protein destabilization, and proteotoxic stress. This review integrates the molecular basis, genetic architecture, long non-coding RNA (lncRNA)-mediated regulation, mechanistic crosstalk, and therapeutic implications of ferroptosis and cuproptosis in cancer. It emphasizes a critical evidence hierarchy: expression association, computational signature construction, metal accumulation, reactive oxygen species (ROS) generation, or reduced viability should not be interpreted as pathway dependency without pathway-defining biochemical endpoints and rescue experiments. The most credible translational opportunities will depend on functional stratification, tumour-selective delivery, and pharmacodynamic confirmation that distinguishes pathway-defined ferroptosis or cuproptosis from nonspecific metal-induced and oxidative cytotoxicity.

## 1. Introduction

Resistance to regulated cell death is a major driver of cancer progression, recurrence, and therapeutic failure. Although apoptosis has historically shaped anticancer drug development, malignant cells frequently evade apoptotic signalling through genetic alterations, lineage plasticity, and metabolic adaptation. This has increased interest in alternative regulated cell-death programs that exploit tumour-specific dependencies rather than relying exclusively on apoptotic competence. Ferroptosis and cuproptosis have emerged as distinct metal-dependent forms of regulated cell death that connect cancer-cell survival to iron and copper homeostasis, redox buffering, mitochondrial metabolism, nutrient availability, and stress-adaptive signalling [1,2,3,4,5,6,7,8,9,10,11,12,13,14,15,16,17,18,19,20,21,22,23,24,25,26,27,28,29,30,31,32,33,34,35,36,37,38,39,40,41,42,43,44,45,46,47,48,49,50,51,52,53,54,55,56,57,58,59,60,61,62,63,64,65,66,67,68,69,70,71,72,73,74,75,76,77,78,79,80,81,82,83,84,85,86,87,88,89,90,91,92,93,94,95,96,97,98,99,100,101,102,103,104,105,106,107,108,109,110,111,112,113].

Ferroptosis is executed through iron-dependent peroxidation of polyunsaturated fatty-acid-containing membrane phospholipids when GPX4-dependent repair and parallel antioxidant systems fail to contain lipid damage. Its sensitivity is shaped by redox-active iron, membrane lipid composition, cystine and glutathione metabolism, and compensatory defence pathways such as FSP1-CoQ, DHODH-CoQ, and GCH1-BH4. Cuproptosis, in contrast, is driven by mitochondrial copper accumulation and copper binding to lipoylated tricarboxylic-acid-cycle proteins, leading to protein aggregation, iron–sulfur cluster destabilization, and proteotoxic stress. Ferroptosis therefore exposes dependence on lipid-peroxide repair, whereas cuproptosis preferentially exposes dependence on oxidative mitochondrial metabolism and protein lipoylation [1,4,5,6].

Despite these distinct execution mechanisms, ferroptosis and cuproptosis are increasingly linked by shared upstream determinants. Glutathione availability, mitochondrial function, metal trafficking, iron–sulfur cluster integrity, NRF2-dependent stress adaptation, and non-coding RNA regulation may all influence whether cancer cells respond to iron-driven lipid peroxidation, copper-triggered mitochondrial proteotoxicity, or overlapping metal-induced injury phenotypes. However, the field remains vulnerable to overinterpretation: mechanistically validated regulators are often discussed alongside computational signatures, correlative expression models, and prognostic scores without sufficient distinction between association and pathway dependency. This gap limits biological interpretation and complicates translation into precision oncology [3,6,8].

Building on previous reviews of copper biology, ferroptosis–cuproptosis crosstalk, and non-coding RNA regulation [6,7,8,77,89], the present review contributes an explicit evidence framework for evaluating mechanistic and translational claims concerning ferroptosis and cuproptosis as metal-dependent forms of regulated cell death. We distinguish pathway-defining regulators from context-dependent modifiers, expression associations, computational signatures, and generic oxidative-stress markers, while separating canonical ferroptosis and cuproptosis from nonspecific metal-induced toxicity. Proposed interactions between the two pathways are further classified as shared susceptibility, directional sensitization, or true co-execution according to whether pathway-specific terminal lesions and independent rescue experiments have been demonstrated. By integrating genetic and lncRNA-mediated regulation with biochemical endpoints, functional rescue, and pharmacodynamic validation, this framework aims to identify which reported cancer vulnerabilities are mechanistically actionable and which remain descriptive or hypothesis-generating.

This narrative review was informed by a targeted literature search of PubMed, Scopus, Web of Science, and Embase, focusing primarily on English-language studies addressing the molecular, genetic, lncRNA-mediated, and therapeutic regulation of ferroptosis and cuproptosis in cancer. Human studies and investigations using patient-derived materials were prioritized when available, while animal and in vitro studies were included when they provided relevant mechanistic or translational evidence. The literature was synthesized narratively to identify pathway-specific mechanisms, points of convergence, differences in evidence strength, unresolved limitations, and clinically relevant opportunities.

This narrative review was informed by a targeted literature search of PubMed, Scopus, Web of Science, and Embase, conducted up to 1 August 2026. The search combined terms related to “ferroptosis,” “cuproptosis,” “copper-dependent cell death,” and “metal-dependent regulated cell death” with cancer-related terms and topic-specific terms covering molecular mechanisms, genetic regulation, long non-coding RNAs, biomarkers, pathway crosstalk, therapeutic targeting, treatment resistance, and translational applications. The search focused primarily on English-language studies. Human studies and investigations using patient-derived materials were prioritized when available, while animal and in vitro studies were included when they provided relevant mechanistic or translational evidence. The literature was synthesized narratively to identify pathway-specific mechanisms, points of convergence, differences in evidence strength, unresolved limitations, and clinically relevant opportunities.

## 2. Mechanistic Framework: Distinct Execution Lesions and Shared Metabolic Constraints

**Figure 1:**
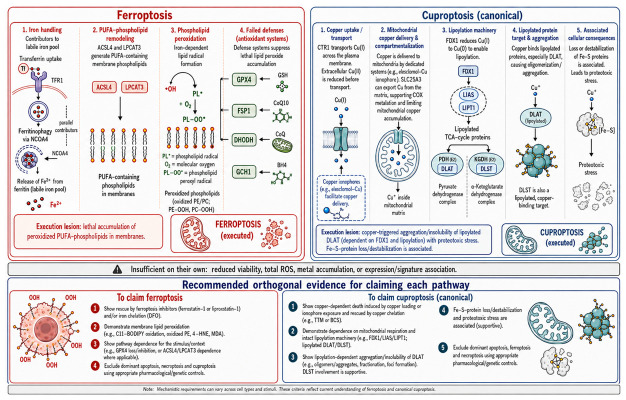
Distinct execution mechanisms and recommended orthogonal evidence for assigning ferroptosis and canonical cuproptosis in cancer studies. Comparative framework for distinguishing ferroptosis from canonical cuproptosis. Ferroptosis is driven by iron-dependent peroxidation of PUFA-containing membrane phospholipids and is regulated by ACSL4, LPCAT3, GPX4, FSP1, DHODH, and GCH1. Canonical cuproptosis involves mitochondrial copper accumulation, dependence on *FDX1*, *LIAS*, and *LIPT1*, and copper-induced aggregation of lipoylated DLAT, with associated Fe–S protein loss and proteotoxic stress. Pathway assignment requires orthogonal pharmacological, biochemical, and genetic evidence; reduced viability, total ROS, metal accumulation, or expression signatures alone are insufficient. Note. GPAI: AI STEM Copilot (Turing Co., Ltd.) was used solely for the technical visual refinement of this author-generated figure. All scientific content, labels, and interpretations were created and verified by the authors.

Cuproptosis and ferroptosis are metal-dependent forms of regulated cell death that reveal distinct, but partially intersecting, vulnerabilities in cancer metabolism. Both pathways become lethal when transition-metal handling is no longer compatible with the metabolic state and stress-buffering capacity of the cell. However, they should not be interpreted as interchangeable variants of metal toxicity. Canonical cuproptosis is initiated when bioavailable copper reaches mitochondria and binds lipoylated enzymes of the tricarboxylic acid (TCA) cycle, promoting their oligomerization, aggregation, iron–sulfur (Fe-S) protein destabilization, and a proteotoxic crisis conditioned by mitochondrial respiratory activity. Ferroptosis, in contrast, is executed through iron-dependent oxidation of polyunsaturated fatty-acid (PUFA)-containing phospholipids when lipid-peroxide formation exceeds the combined capacity of GPX4-dependent repair and parallel radical-trapping systems. Thus, cuproptosis is centred on mitochondrial protein lipoylation and proteostasis, whereas ferroptosis is centred on membrane lipid composition and phospholipid peroxidation [1,2,3,5,6,9,10,11,12,13,14,15,16]. Figure 1 integrates these distinct execution lesions with the principal molecular determinants and the orthogonal evidence required for pathway assignment.

Major candidate biomarkers can be divided into markers of pathway susceptibility and more proximal indicators of pathway engagement. For ferroptosis, TFRC and ACSL4 are commonly used susceptibility-associated markers, whereas SLC7A11 and GPX4 reflect the strength of the canonical cystine–glutathione–GPX4 defence axis. Expansion of the labile Fe(II) pool and, more specifically, the accumulation of oxidized PUFA-containing phospholipids, including oxidized arachidonoyl- and adrenoyl-phosphatidylethanolamine species, provide more direct evidence of ferroptotic execution. For cuproptosis, FDX1, LIAS, LIPT1, and the abundance and lipoylation status of DLAT and DLST indicate pathway competence, whereas mitochondrial copper accumulation, aggregation of lipoylated proteins—particularly DLAT—and loss of Fe–S-cluster proteins provide more proximal evidence of pathway engagement. However, none of these biomarkers should be interpreted in isolation, and no single clinically validated biomarker currently establishes ferroptosis or cuproptosis without pathway-specific biochemical and rescue evidence [1,2,3,5,6,9,10,11,12,13,14,15,16].

### 2.1. Cuproptosis: Mechanistic Overview

Copper is an essential transition metal that supports mitochondrial respiration, antioxidant defence, iron metabolism, extracellular-matrix maturation, neurotransmitter synthesis, and intracellular signalling. Its chemical versatility is also potentially hazardous; therefore, mammalian cells maintain a very small bioavailable copper pool through controlled uptake, thiol-dependent sequestration, chaperone-mediated trafficking, organellar delivery, and export. Copper entering through transport systems such as SLC31A1 is rapidly buffered by glutathione or metallothioneins, delivered by specialized chaperones to cuproproteins, or redistributed and exported by ATP7A and ATP7B. Cuproptosis becomes possible when these buffering systems are overwhelmed, genetically impaired, or pharmacologically bypassed. Copper ionophores such as elesclomol are particularly relevant because they increase intracellular and mitochondrial copper delivery, converting an essential micronutrient into a selective metabolic toxicant. Importantly, the relevant variable is not total cellular copper, but the oxidation state, ligand environment, and subcellular distribution of the bioavailable copper fraction [1,6,9,11,12,13,15,16].

The defining biochemical feature of canonical cuproptosis is the direct interaction of copper with lipoylated mitochondrial proteins. Protein lipoylation is a conserved post-translational modification in which a lipoic-acid cofactor is covalently attached to selected mitochondrial multienzyme complexes. In human cells, the best-characterized cuproptosis-relevant substrates include dihydrolipoamide S-acetyltransferase (DLAT), encoded by *DLAT*, in the pyruvate dehydrogenase complex, and dihydrolipoamide S-succinyltransferase (DLST), encoded by *DLST*, in the alpha-ketoglutarate dehydrogenase complex. These enzymes connect glycolytic carbon entry, glutamine-derived anaplerosis, acetyl-CoA generation, and TCA-cycle flux; consequently, their abundance and lipoylation state reflect the extent to which a cell relies on oxidative mitochondrial metabolism. The mitochondrial lipoylation program involving FDX1, LIAS, LIPT1, and DLD determines whether a cell possesses a sufficiently active lipoylated proteome to support canonical cuproptotic execution [1,6,9,11,12,13,15,16].

*FDX1* appears to occupy an upstream regulatory position because its loss decreases mitochondrial protein lipoylation and attenuates copper-ionophore-induced death in human cancer cells. FDX1 has also been proposed to facilitate Cu(II)-to-Cu(I) reduction, which may increase the copper fraction capable of engaging mitochondrial targets; however, the relative contribution of copper reduction versus lipoylation control remains incompletely resolved. Disruption of LIAS reduces the generation of lipoylated mitochondrial proteins and confers resistance, while perturbation of *LIPT1*, *DLD*, *DLAT*, or *DLST* alters the availability or function of cuproptosis-sensitive metabolic substrates. These observations establish a key mechanistic point: copper accumulation is not sufficient for canonical cuproptosis when the lipoylated mitochondrial proteome is absent, inactive, or functionally suppressed. Conversely, cells with high respiratory flux and abundant lipoylated TCA-cycle enzymes may become vulnerable to relatively modest increases in mitochondrial copper [1,6,9,11,12,13,15,16].

Following mitochondrial delivery, copper binding to lipoylated TCA-cycle proteins promotes abnormal oligomerization and the formation of insoluble aggregates, with lipoylated DLAT representing the most extensively characterized substrate. This process converts a physiological cofactor modification required for central carbon metabolism into a proteotoxic liability. Aggregation of lipoylated proteins is accompanied by reduced abundance of Fe-S-cluster proteins, which can amplify mitochondrial dysfunction by impairing electron transport, TCA-cycle metabolism, iron sensing, DNA synthesis and repair, and redox reactions. Nevertheless, the causal hierarchy between lipoylated-protein aggregation and Fe-S-protein loss remains unresolved. Fe-S-protein depletion should therefore be interpreted as part of the cuproptotic phenotype, not as a stand-alone diagnostic marker [1,6,9,11,12,13,15,16].

The requirement for an active lipoylated proteome explains the close relationship between cuproptosis and mitochondrial respiration. Cells maintained under conditions that favour oxidative phosphorylation are generally more sensitive to copper ionophores than cells that rely predominantly on glycolysis, whereas hypoxia, restriction of mitochondrial pyruvate entry, or selected electron-transport-chain perturbations can reduce sensitivity. This relationship should not be simplified to ATP production alone. Respiratory metabolism sustains TCA-cycle flux and preserves the functionally engaged lipoylated enzymes that serve as copper-binding substrates. For this reason, cuproptosis has attracted interest as a potential vulnerability of oxidative tumours or therapy-resistant subpopulations with increased mitochondrial dependence. At present, however, oxidative phosphorylation dependence has not been validated prospectively as a clinical biomarker, and susceptibility will probably require integrated assessment of copper trafficking, mitochondrial copper delivery, protein lipoylation, metabolic flux, and stress-response capacity [6,9,11,12,13,15,16].

A final point is essential for experimental classification. Canonical cuproptosis is not equivalent to nonspecific oxidative copper toxicity. Excess copper can generate reactive oxygen species, oxidize thiols, deplete glutathione, impair mitochondrial membranes, and induce apoptosis, ferroptosis, necrosis, or mixed injury under certain conditions. These effects do not by themselves demonstrate cuproptosis. Classification should be supported by copper dependence, mitochondrial respiratory or lipoylation dependence, aggregation of lipoylated mitochondrial proteins, and accompanying Fe-S-protein alterations. ROS may modify the magnitude or kinetics of injury, particularly when copper concentrations are high or antioxidant buffering is impaired, but iron-dependent phospholipid peroxidation is not the defining biochemical engine of canonical cuproptosis [1,6,9,11,12,13,15,16].

### 2.2. Ferroptosis: Mechanistic Overview

Ferroptosis is defined by the lethal accumulation of iron-dependent phospholipid peroxides in cellular membranes. Its execution depends on three interacting modules: the size and accessibility of the redox-active iron pool, the abundance and localization of peroxidizable PUFA-containing phospholipids, and the capacity of enzymatic and non-enzymatic systems to reduce lipid hydroperoxides or terminate lipid-radical chain reactions. Ferroptosis therefore cannot be defined by bulk ROS accumulation alone. Many oxidative insults increase cytosolic or mitochondrial ROS without producing the spatially organized phospholipid-peroxidation pattern required for ferroptotic execution. The decisive lesion is the failure to control PUFA-phospholipid oxidation within susceptible membrane domains [2,3,5].

The intracellular labile iron pool supplies the redox-active iron required for ferroptotic lipid oxidation. Transferrin-bound Fe(III) is internalized through transferrin receptor 1, reduced in endosomes, and transported into the cytosol as Fe(II), where it can be used as an enzymatic cofactor, stored in ferritin, incorporated into heme or Fe-S clusters, or exported through ferroportin. Expansion of the labile Fe(II) pool increases the probability of Fenton-type reactions and can support enzymatic lipid oxidation through lipoxygenases and other iron-dependent oxidoreductases. Ferritin and NCOA4-mediated ferritinophagy are therefore central regulators of ferroptosis sensitivity: ferritin limits uncontrolled iron reactivity, whereas ferritinophagy releases stored iron and can expand the labile pool. Still, iron accumulation is permissive rather than independently sufficient. Cells with substantial labile iron may resist ferroptosis if they lack peroxidizable membrane substrates or maintain strong lipid-peroxide repair systems [2,3,5,17].

The second requirement is a membrane lipid landscape capable of sustaining radical propagation. PUFAs are particularly susceptible to peroxidation because bis-allylic carbon-hydrogen bonds are easily abstracted during radical reactions. However, ferroptosis sensitivity depends less on the total amount of free PUFAs than on their activation and incorporation into membrane phospholipids. ACSL4 generates PUFA-acyl-CoA derivatives, especially from arachidonic and adrenic acids in many cancer contexts, while LPCAT3 contributes to their incorporation into phospholipids through membrane-remodelling reactions. In contrast, enrichment in monounsaturated fatty acids, sequestration of PUFAs in neutral lipids, altered phospholipid remodelling, or removal of oxidized acyl chains can reduce ferroptosis-relevant substrates. Susceptibility is therefore determined by the chemical identity, phospholipid class, subcellular localization, and accessibility of PUFA-containing membrane species [2,3,5].

The canonical ferroptosis-defence pathway is organized around cystine uptake, glutathione synthesis, and GPX4-dependent detoxification of phospholipid hydroperoxides. System $x_{c}^{\text{-}}$, composed of SLC7A11 and SLC3A2, imports extracellular cystine in exchange for intracellular glutamate. Cystine is then reduced to cysteine and incorporated into glutathione, which serves as the reducing substrate for GPX4. GPX4 converts phospholipid hydroperoxides into non-propagating phospholipid alcohols, preventing isolated oxidation events from becoming self-amplifying radical chains. Ferroptosis can be initiated upstream by cystine deprivation, *SLC7A11* suppression, or disruption of glutathione biosynthesis, or downstream by direct GPX4 inhibition. These routes converge on lipid-peroxide accumulation but differ in kinetics and associated metabolic consequences, meaning that resistance mechanisms identified under one induction condition cannot automatically be generalized to all forms of ferroptosis [2,3,5].

GPX4 is central, but it is not the only barrier to ferroptosis. FSP1, encoded by *AIFM2*, uses NAD(P)H to reduce coenzyme Q to ubiquinol at cellular membranes, generating a lipophilic radical-trapping antioxidant independently of glutathione. In the inner mitochondrial membrane, DHODH couples pyrimidine metabolism to coenzyme Q reduction and provides compartmentalized protection that can compensate for low mitochondrial GPX4 activity. The GCH1-tetrahydrobiopterin pathway supplies an additional antioxidant and lipid-remodelling mechanism. The relative contribution of these systems varies among cancer lineages and metabolic states, explaining why equivalent GPX4 expression or glutathione depletion does not produce uniform sensitivity. Ferroptosis occurs when the combined capacity of GPX4-dependent detoxification, quinone-based radical trapping, biopterin-dependent protection, membrane repair, and iron sequestration is insufficient to contain phospholipid oxidation [2,3,4,5].

### 2.3. Key Differences, Similarities, and Mechanistic Intersections

The most useful distinction between cuproptosis and ferroptosis is the molecular substrate on which lethal damage accumulates. In cuproptosis, copper engages a restricted group of lipoylated mitochondrial enzymes and converts them into abnormal oligomeric and aggregated species, leading to mitochondrial proteotoxic collapse and Fe-S-protein destabilization. In ferroptosis, redox-active iron supports radical-chain oxidation of PUFA-containing membrane phospholipids, and death occurs when lipid hydroperoxides exceed the capacity of GPX4 and parallel defence systems. The classification of these pathways should therefore be based on their defining execution lesions, not on secondary consequences shared by many metal-induced injuries, such as ROS generation, mitochondrial dysfunction, or loss of viability [1,2,3,5,6,9,10,11,12,13,14,15,16].

Copper and iron biology also intersect, which is why experimental interventions may produce overlapping injury mechanisms or concurrent features of more than one regulated cell-death pathway. Copper can influence Fe-S-cluster stability, iron handling, glutathione availability, mitochondrial metabolism, and antioxidant capacity. Conversely, iron-dependent oxidative stress can alter mitochondrial function, metal trafficking, and redox-buffering systems. These connections are biologically meaningful, but they also create a classification problem: metal accumulation, ROS generation, mitochondrial damage, or reduced viability are not sufficient to assign a pathway-specific mechanism. This issue is especially relevant for copper-containing nanoparticles, disulfiram–copper combinations, iron-modulating compounds, and multifunctional redox-active agents, which may simultaneously perturb copper homeostasis, iron availability, thiol metabolism, mitochondrial function, and membrane lipid oxidation [1,2,3,5,6,9,10,11,12,13,14,15,16].

Future mechanistic studies should therefore apply pathway-specific validation criteria. Cuproptosis should be supported by copper dependence, mitochondrial respiratory or lipoylation dependence, aggregation of lipoylated mitochondrial proteins, and accompanying Fe-S-protein alterations. Ferroptosis should be supported by iron-dependent phospholipid peroxidation and appropriate genetic or pharmacological rescue, including suppression by iron chelation, radical-trapping antioxidants, inhibition of phospholipid peroxidation, or restoration of ferroptosis-defence pathways. Using these criteria will help distinguish canonical ferroptosis and cuproptosis from nonspecific metal-induced cytotoxicity and will make it possible to determine when the two pathways operate independently, cooperate, or coexist within a broader metal-induced cellular-injury response in cancer [1,2,3,5,6,9,10,11,12,13,14,15,16].

## 3. Genetic Architecture of Cuproptosis

Cuproptosis is governed by a restricted mitochondrial gene network that integrates copper availability, protein lipoylation, and oxidative metabolism. In the canonical model, excess intracellular copper binds to lipoylated components of the tricarboxylic acid (TCA) cycle, promotes lipoylated-protein aggregation, destabilises iron–sulfur (Fe-S) cluster proteins, and induces proteotoxic stress [1,18]. Genetic susceptibility to this process is therefore determined not only by cellular copper uptake and export, but also by the integrity of the mitochondrial lipoylation machinery and the metabolic state of the tumour cell.

The cuproptosis network should be interpreted at several levels. Positive regulators identified in the canonical CRISPR-screen framework include FDX1, LIAS, LIPT1, DLD, DLAT, PDHA1, and PDHB, whereas MTF1, GLS, and CDKN2A have been described as negative regulators of cuproptotic susceptibility [1,18]. In parallel, SLC31A1/CTR1, ATP7A, and ATP7B influence intracellular copper availability and act as copper-homeostasis modifiers. Distinguishing core pathway genes from copper transporters and broader metabolic regulators is therefore essential when interpreting their roles in cancer, as summarized in Table 1.

### 3.1. Core Cuproptosis Genes

Ferredoxin 1 (FDX1) is a central determinant of canonical cuproptosis. FDX1 is a mitochondrial Fe-S protein with established roles in electron transfer. In the context of the copper ionophore elesclomol, FDX1 reduces ionophore-bound Cu(II) to Cu(I), facilitating mitochondrial copper release [19]. FDX1 also promotes mitochondrial protein lipoylation by directly binding lipoyl synthase (LIAS) and supporting its functional interaction with the lipoyl carrier protein GCSH [20]. Accordingly, FDX1 deficiency reduces mitochondrial protein lipoylation and confers resistance to copper-ionophore-induced cell death [1,20]. FDX1 should therefore be described as a central determinant rather than as a universal intracellular copper-reduction enzyme.

The lipoylation machinery is indispensable for canonical cuproptosis. LIAS inserts sulfur atoms during lipoic-acid biosynthesis, whereas lipoyltransferase 1 (LIPT1) supports transfer of the lipoyl moiety to selected mitochondrial proteins. Protein lipoylation is restricted to a small group of mitochondrial enzymes involved in oxidative metabolism, including DLAT, DLST, DBT, and GCSH [21,22]. This restricted substrate range helps explain why cuproptosis is selective for cells with active mitochondrial metabolism rather than a nonspecific consequence of copper overload.

Among the lipoylated proteins, dihydrolipoamide S-acetyltransferase (DLAT), the E2 component of the pyruvate dehydrogenase complex, is the best-characterized cuproptotic effector. Copper binding promotes DLAT oligomerisation and aggregation, linking pyruvate metabolism directly to proteotoxic stress [1,23]. DLST, DBT, and GCSH contribute to the broader lipoylation-dependent metabolic context, but the experimental depth is not identical for each protein. They should therefore not be presented as equivalent to DLAT or as equally validated primary copper-binding targets.

Recent structural work has refined the role of FDX1. Deep mutational scanning identified two solvent-exposed residues, D136 and D139, within alpha-helix 3 as essential for both mitochondrial protein lipoylation and cuproptosis. Charge-reversal mutations at these sites abolished both cellular phenotypes despite preserving enzymatic activity in vitro [24]. The same study proposed dihydrolipoamide dehydrogenase (DLD) as an alternative upstream electron donor for FDX1, linking mitochondrial electron transfer to cuproptotic competence. *PDHA1* and *PDHB*, which encode the E1 alpha and beta subunits of the pyruvate dehydrogenase complex, further connect the pathway to pyruvate entry into the TCA cycle [25].

Negative regulators should also be acknowledged. MTF1, GLS, and CDKN2A were identified within the canonical screening framework as genes whose loss increases sensitivity to cuproptotic stress [1,18]. Although their precise roles remain less well defined than those of FDX1, LIAS, and DLAT, their inclusion is important because it demonstrates that cuproptosis is governed by both permissive and protective genetic programs. Table 1 provides a comparative overview of these regulators and their proposed effects on cuproptotic susceptibility.

### 3.2. Copper Homeostasis Genes

Copper-homeostasis genes modify the intracellular copper pool available to trigger cuproptosis. *SLC31A1*, also known as copper transporter 1 (CTR1), is a high-affinity cellular copper importer, whereas *ATP7A* and *ATP7B* contribute to intracellular trafficking and copper export [6,26,27]. This balance is relevant to cancer cells because excessive copper accumulation can increase susceptibility to cuproptosis, while efficient export may provide a protective mechanism.

ATP7A and ATP7B counterbalance copper uptake by supporting intracellular redistribution and export. Under basal conditions, these ATPases predominantly localize to the trans-Golgi network; when intracellular copper rises, they traffic towards vesicular compartments and the plasma membrane to facilitate copper removal [6,26]. Their contribution is likely to vary by tissue context because ATP7A and ATP7B differ in expression patterns and physiological roles. In a tumour-cell setting, the most relevant question is not their systemic function, but whether transporter activity limits the intracellular copper pool available to trigger canonical cuproptosis. A recent mechanistic study in *KRAS*-mutant lung adenocarcinoma provides a particularly relevant therapeutic example: elesclomol increased copper accumulation and cuproptosis by promoting OSTM1-dependent ubiquitination and degradation of ATP7A. The effect was evaluated in tumour cells, organoids, and mouse models [28]. This evidence is more compelling than broad correlative associations and supports copper export as a tractable therapeutic vulnerability in selected tumour contexts, with the relevant molecular targets and supporting findings detailed in Table 1.

### 3.3. Genetic Alterations in Cancer

The expression and genomic alteration patterns of cuproptosis-related genes vary across tumour types. Pan-cancer studies have reported differential expression, copy-number changes, mutations, and context-dependent survival associations involving copper-metabolism and cuproptosis-related gene sets [27,29]. These analyses suggest that the pathway is not uniformly activated or suppressed across cancer. Instead, the biological consequence of an alteration depends on tumour lineage, metabolic state, and the balance between copper uptake, export, and mitochondrial lipoylation.

These findings should be interpreted cautiously. Transcriptomic abundance, promoter methylation, immune-cell deconvolution, and drug-sensitivity correlations are valuable for prioritising candidate genes, but they do not establish causality. Copy-number gain or increased messenger-RNA expression does not necessarily indicate increased pathway activity, because the cuproptotic response also depends on protein abundance, subcellular localization, copper availability, and mitochondrial metabolic competence. Conversely, low expression of a single pathway component may be compensated by other elements of the network. The most persuasive genetic alterations are therefore those supported by mechanistic experiments showing that perturbation changes intracellular copper handling, mitochondrial lipoylation, or response to copper-ionophore treatment as presented in Table 1.

### 3.4. Metabolic Context

Cuproptosis sensitivity is metabolically encoded. Cells that rely strongly on mitochondrial respiration are more susceptible to copper-ionophore-induced death than cells that rely predominantly on glycolysis [1]. This dependence is consistent with the restricted localization of protein lipoylation to mitochondrial enzymes involved in carbon flux. The pyruvate dehydrogenase complex is particularly relevant because it links glycolysis to acetyl-CoA production and the TCA cycle through PDHA1, PDHB, and DLAT.

This metabolic framework has two implications for cancer biology. First, tumours with an oxidative phosphorylation-dependent phenotype may be more vulnerable to cuproptosis-inducing strategies. Second, glycolytic adaptation may provide resistance by reducing dependence on lipoylated mitochondrial enzymes. This distinction is particularly relevant in heterogeneous tumours, where metabolically distinct subclones may differ in sensitivity even when they share the same driver mutation. It also helps explain why the therapeutic activity of copper ionophores cannot be predicted solely from total cellular copper content, as outlined in Table 1.

## 4. Genetic Architecture of Ferroptosis and Interfaces with Cuproptosis

Ferroptosis is not governed by a single execution gene. In cancer cells, susceptibility emerges from the combined activity of genetic modules that control redox-active iron, the availability of peroxidizable membrane phospholipids, and the antioxidant systems that repair or intercept lipid-peroxidation reactions. This section therefore prioritizes genes with clear mechanistic relevance rather than presenting an exhaustive catalogue of every reported ferroptosis-associated transcript. The most informative framework is modular: iron-handling genes establish the labile iron pool; lipid-remodelling genes define membrane susceptibility; antioxidant-defence genes determine the capacity to suppress phospholipid peroxidation; and oncogenes or tumour suppressors reshape these modules through broader metabolic and transcriptional programs. This network perspective is also central to the present review, as several ferroptosis-regulating genes intersect indirectly with cuproptosis through shared control of metal homeostasis, glutathione availability, mitochondrial function, Fe-S-cluster biology, and stress-adaptive signalling [2,3,30,31], with these points of convergence mapped in Table 2.

### 4.1. Iron Metabolism Genes and the Labile Iron Pool

The ferroptotic threshold is strongly influenced by genes that regulate iron uptake, storage, mobilization, and export. The relevant determinant is not total cellular iron, but the fraction of redox-active Fe(II) available to support phospholipid peroxidation. TFRC, which encodes transferrin receptor 1, is a major positive regulator because it mediates transferrin-bound iron uptake. After endocytosis, STEAP3 reduces Fe(III) to Fe(II), and DMT1, encoded by SLC11A2, transports Fe(II) into the cytosol, thereby replenishing the labile iron pool. Increased TFRC activity often promotes ferroptosis sensitivity, whereas suppression of this axis can reduce iron availability and attenuate ferroptotic death. However, TFRC expression alone should not be treated as a universal biomarker, because iron storage, export, mitochondrial iron use, and antioxidant capacity can all modify the final phenotype [31,32,33,34,35].

Ferritin genes counterbalance iron uptake. The FTH1 and FTL genes encode the heavy and light ferritin chains, respectively, which assemble into a heteropolymeric complex that sequesters intracellular iron and limits its participation in iron-dependent lipid oxidation. The FTH1 subunit provides ferroxidase activity, converting Fe(II) into the less reactive Fe(III) state, whereas FTL facilitates ferritin assembly, iron nucleation, and mineralization. Loss of or reduction in ferritin can increase the availability of catalytic iron and sensitize cells to ferroptosis. Conversely, NCOA4 promotes ferritinophagy by delivering ferritin to lysosomes for degradation, thereby releasing stored iron and expanding the labile iron pool. Ferroportin, encoded by SLC40A1, exerts the opposite effect by exporting iron and limiting intracellular iron accumulation. Together, TFRC, STEAP3, SLC11A2, FTH1, FTL, NCOA4, and SLC40A1 form a core iron-handling module that determines whether cancer cells possess sufficient accessible iron to sustain lethal phospholipid peroxidation [30,31,36,37].

Ferritin abundance is not determined solely by iron storage and NCOA4-mediated degradation but is also shaped by transcriptional and post-transcriptional regulatory mechanisms. Promoter-level control of FTH1 has been directly connected to ferroptosis in osteosarcoma cells, in which TFEB binding to the FTH1 promoter was confirmed using luciferase-reporter and chromatin-immunoprecipitation assays. In this setting, TFEB-mediated transcriptional repression of FTH1 was associated with enhanced lysosomal biogenesis, ferritinophagy, Fe(II) accumulation, and ferroptotic death [114]. FTH1 expression is also regulated by non-coding RNAs and RNA-binding proteins. In lung cancer cells, lncRNA H19 acted as a competing endogenous RNA for miR-19b-3p, maintaining FTH1 expression and limiting curcumenol-induced ferroptosis [72]. In colorectal cancer cells, the RNA-binding protein RSL1D1 interacted with the 3′ untranslated region of FTH1 and stabilized its transcript; RSL1D1 depletion reduced FTH1 abundance, increased intracellular Fe(II), and promoted ferroptosis [115]. These findings demonstrate that promoter activity, microRNA competition, and transcript stability can independently modify ferritin abundance and thereby alter the ferroptotic threshold.

An additional regulatory layer is provided by transcribed FTH1 pseudogenes. FTH1P3 is not a transcriptionally silent genomic remnant: its transcript has been detected across multiple tissues and cell lines, and its expression increases during cellular differentiation. Sequence analysis has also identified a highly homologous region within the 3′ untranslated regions of FTH1P3 and FTH1 containing several predicted shared microRNA-response elements, suggesting the potential for microRNA-mediated regulatory crosstalk [116]. Consistent with a functional non-coding role, FTH1P3 has been experimentally shown to interact with the tumor-suppressive miRNA miR-145 in cervical cancer cells, thereby influencing proliferation, migration, invasion, and apoptosis [117].

More broadly, experimental evidence from prostate cancer has established that several other FTH1 pseudogenes, including FTH1P2, FTH1P8, FTH1P11, and FTH1P16, can participate in a gene–pseudogene–microRNA network. These pseudogene transcripts competed for shared microRNAs, altered FTH1 protein abundance and intracellular iron levels, and affected tumor growth in vitro and in vivo [118]. This study provides direct proof that pseudogene-derived transcripts can regulate ferritin biology through competing endogenous RNA mechanisms. However, it did not investigate FTH1P3 specifically. Therefore, although the transcriptional activity, shared predicted microRNA-response elements, and demonstrated microRNA-binding capacity of FTH1P3 support its possible involvement in ferritin regulation, a direct FTH1P3–FTH1 regulatory axis controlling ferroptosis has not yet been conclusively established.

Collectively, these findings expand the conventional view of ferritin as a passive iron-storage complex. Transcription factors, RNA-binding proteins, microRNAs, lncRNAs, and pseudogene-derived transcripts can alter FTH1 abundance and consequently shift the balance between safely sequestered iron and the redox-active labile iron pool. By modifying iron availability, ROS generation, and the substrate supply for phospholipid peroxidation, these mechanisms may enhance or suppress ferroptosis in a cell- and context-dependent manner. The effects of FTH1P3 on ferroptosis should therefore be regarded as mechanistically plausible but still requiring direct experimental validation.

This iron module is also relevant to a combined ferroptosis–cuproptosis framework. Cuproptosis is accompanied by destabilization of iron–sulfur-cluster proteins, while ferroptosis depends on iron mobilization and redox-active iron chemistry. These mechanisms are not identical, but they converge on mitochondrial metal handling and Fe–S-cluster integrity. Therefore, alterations in iron metabolism genes may influence whether a therapeutic intervention produces canonical ferroptosis, canonical cuproptosis, or overlapping metal-induced injury that does not yet satisfy the criteria for either pathway or for true co-execution [1,2,3]. The principal iron-handling genes and their effects on ferroptosis susceptibility are summarized in Table 2.

### 4.2. Lipid-Remodelling Genes and Membrane Susceptibility

Iron availability is permissive but insufficient for ferroptosis unless membranes contain oxidizable substrates. Ferroptosis is executed through the peroxidation of phospholipids enriched in polyunsaturated fatty acids, particularly arachidonic acid and adrenic acid. ACSL4 is one of the most robust genetic determinants of this lipid state because it converts free polyunsaturated fatty acids into acyl-CoA derivatives that can be incorporated into membrane phospholipids. LPCAT3 then contributes to Lands-cycle remodelling by inserting these activated acyl chains into phospholipid species, especially phosphatidylethanolamines and phosphatidylcholines. Genetic or lipidomic studies showing that loss of *ACSL4* or *LPCAT3* reduces PUFA-containing phospholipids and confers resistance to GPX4 inhibition established this axis as a central determinant of ferroptotic competence [38,39,40,41,42].

Nevertheless, ACSL4 and LPCAT3 should not be interpreted as absolute requirements in every cellular context. Ferroptosis sensitivity can vary according to the inducer, tumour lineage, and lipid supply. Upstream PUFA-biosynthesis genes, including *ELOVL5*, *ELOVL2*, *FADS1*, and *FADS2*, influence the availability of arachidonic- and adrenic-acid substrates, and epigenetic silencing of these genes can reduce ferroptotic competence in selected cancer models. Downstream, ALOX12 and ALOX15 may amplify phospholipid oxidation, but their contribution is context-dependent and may be less critical in settings driven by direct GPX4 inhibition. In parallel, POR and CYB5R1 can support ALOX-independent lipid peroxidation, indicating that ferroptotic membrane damage can arise through several distinct redox architectures [38,43,44], as delineated in Table 2.

### 4.3. Antioxidant-Defence Genes and Compensatory Ferroptosis Resistance

The third major genetic module determines whether lipid peroxides are repaired, neutralized, or allowed to propagate. The canonical pathway begins with system $x_{c}^{\text{-}}$, composed of SLC7A11 and SLC3A2, which imports cystine in exchange for glutamate. Intracellular cystine is reduced to cysteine and incorporated into glutathione through enzymes encoded by *GCLC*, *GCLM*, and *GSS*. GPX4 then uses reduced glutathione to convert phospholipid hydroperoxides into non-propagating phospholipid alcohols, while GSR regenerates reduced glutathione in an NADPH-dependent manner. Disruption of *SLC7A11*, glutathione synthesis, or GPX4 lowers the antioxidant threshold and sensitizes cells to ferroptosis. Direct GPX4 inhibition is mechanistically distinct from upstream cystine deprivation because it disables phospholipid repair even when glutathione remains present [2,3,45,46].

GPX4 is central, but it is not the only genetically encoded barrier to ferroptosis. *AIFM2* encodes FSP1, a membrane-associated oxidoreductase that reduces ubiquinone to ubiquinol and can also reduce vitamin K to a radical-trapping hydroquinone. DHODH provides a spatially distinct mitochondrial defence by reducing ubiquinone within the inner mitochondrial membrane during de novo pyrimidine synthesis, thereby limiting mitochondrial lipid peroxidation. GCH1 promotes synthesis of tetrahydrobiopterin, another lipophilic antioxidant, while DHFR contributes to biopterin recycling. These pathways explain why GPX4 inhibition does not produce uniform responses across cancer types: resistance may be maintained by FSP1, DHODH, GCH1-BH4, NADPH-generating pathways, or lipid-remodelling programs that reduce the burden of oxidizable substrates [4,5,47,48,49].

From a translational perspective, antioxidant-defence genes should be interpreted as a network rather than as isolated markers. High SLC7A11 may predict dependence on cystine uptake, but it can also create vulnerability when glucose metabolism and NADPH production are impaired because imported cystine must be reduced to cysteine. Similarly, GPX4 expression may underestimate ferroptosis resistance if FSP1, DHODH, or GCH1 are strongly active. A more useful predictive model would integrate SLC7A11, GPX4, *AIFM2*/FSP1, DHODH, GCH1, ACSL4, iron-handling genes, and functional lipid-peroxidation readouts. This same antioxidant network may also affect cuproptosis: glutathione, metallothioneins, and NRF2-regulated detoxification programs can buffer copper and reduce the labile copper pool, potentially limiting copper-induced mitochondrial proteotoxicity while simultaneously suppressing ferroptosis [1,2,3,5], as illustrated in Table 2.

### 4.4. Oncogenes and Tumour Suppressors: Context-Dependent Control

Oncogenes and tumour suppressors regulate ferroptosis by reshaping the metabolic environment in which the core iron, lipid, and antioxidant modules operate. *TP53* provides the clearest example of context dependence. p53 can promote ferroptosis by repressing *SLC7A11*, reducing cystine uptake, weakening glutathione-dependent GPX4 activity, and facilitating ALOX12-associated lipid peroxidation. However, p53 can also delay ferroptosis through *CDKN1A*/p21-mediated slowing of cellular metabolism or through cytoplasmic inhibition of DPP4-NOX1-dependent membrane ROS production. The net effect depends on p53 mutation type, acetylation state, cellular lineage, nutrient context, and the ferroptosis inducer used. Therefore, *TP53* status alone is unlikely to function as a universal predictive biomarker. It should be interpreted together with *SLC7A11*, *CDKN1A*, DPP4, ALOX12, PLA2G6, GPX4 activity, and lipid-peroxidation measurements [2,3,50,51,52,53].

The *KEAP1-NFE2L2* axis generally acts in the opposite direction by establishing a coordinated ferroptosis-resistant state. NRF2, encoded by *NFE2L2*, activates genes involved in cystine uptake, glutathione synthesis, NADPH utilization, electrophile detoxification, iron storage, iron export, and FSP1-dependent radical trapping. Functional KEAP1 restrains this program by promoting NRF2 ubiquitination and degradation; *KEAP1* loss or activating *NFE2L2* mutations convert a transient stress response into a constitutive survival program. This is particularly relevant in non-small-cell lung cancer, where *KEAP1*-inactive tumours can upregulate FSP1 and resist both ferroptosis and radiation-associated lipid peroxidation. Importantly, functional NRF2 activation may occur even in the absence of KEAP1 or NFE2L2 mutations, through mechanisms such as reduced KEAP1 abundance, p62/SQSTM1-mediated sequestration, DPP9-associated NRF2 stabilization, and other upstream regulatory alterations [2,3,54,55], all of which are incorporated into the regulatory framework presented in Table 2.

### 4.5. Genetic Interfaces Between Ferroptosis and Cuproptosis

Although the genes discussed above are usually framed as ferroptosis regulators, several of them may also influence cuproptosis susceptibility indirectly. The most important shared nodes are not the terminal lesions themselves, which remain distinct, but the cellular systems that determine whether metal-dependent stress becomes lethal. Ferroptosis is executed by iron-dependent phospholipid peroxidation, whereas canonical cuproptosis is driven by copper binding to lipoylated mitochondrial proteins, aggregation of these proteins, Fe-S-protein destabilization, and mitochondrial proteotoxic stress. However, both pathways are shaped by metal buffering, glutathione availability, mitochondrial metabolism, and stress-response programs [1,2,3,6].

Future biomarker panels should therefore avoid single-gene classification and instead combine ferroptosis-relevant genes with cuproptosis determinants such as *FDX1*, *LIAS*, *LIPT1*, *DLAT*, *DLST*, *SLC31A1*, *ATP7A*, and *ATP7B*. For ferroptosis, the minimal informative panel should include iron-handling genes, PUFA-remodelling genes, and antioxidant-defence genes. For cuproptosis, it should include copper import/export, mitochondrial respiration, protein lipoylation, and Fe-S-cluster integrity. Integrating these layers would allow investigators to distinguish tumours that are primarily ferroptosis-prone, cuproptosis-prone, resistant to both, or vulnerable to rational co-induction. Such a network-based approach is better aligned with precision oncology than reliance on isolated markers such as TFRC, ACSL4, SLC7A11, GPX4, or FDX1 alone [1,2,3,6], as reflected in the integrated gene-level framework provided in Table 2.

## 5. lncRNA-Mediated Regulation of Cuproptosis

Long non-coding RNAs (lncRNAs) are emerging as context-dependent regulators of cuproptosis and copper-associated tumour biology. Cuproptosis is a copper-dependent form of regulated cell death that is mechanistically distinct from apoptosis and ferroptosis. Excess intracellular copper binds to lipoylated components of the tricarboxylic acid (TCA) cycle, promotes lipoylated-protein aggregation, destabilises iron–sulfur cluster proteins, and induces proteotoxic stress. Because this process depends strongly on mitochondrial respiration, cuproptosis sensitivity is determined not only by intracellular copper availability but also by the metabolic state of the tumour cell [1].

Compared with lncRNA-mediated ferroptosis regulation, the cuproptosis field remains at an earlier stage of mechanistic development. The available literature includes a limited number of direct molecular studies, several functional associations, predicted regulatory networks, and a larger body of retrospective prognostic signatures. These categories should not be interpreted interchangeably. In this section, the term cuproptosis-regulating lncRNA is reserved for transcripts supported by functional mechanistic evidence. The term cuproptosis-associated lncRNA refers to transcripts linked to cuproptosis-related genes through expression analysis, co-expression analysis, or preliminary functional experiments. Cuproptosis-related lncRNA signatures are retrospective models derived from transcriptomic datasets, with representative examples presented in Table 3.

A direct transcriptional mechanism has been described in colorectal cancer (CRC), where *PVT1* promotes cuproptosis through epigenetic activation of ferredoxin 1 (*FDX1*). *PVT1* directly binds to the *FDX1* promoter, increases H3K27 acetylation, and recruits the transcription factor SF1, thereby increasing *FDX1* transcription. The study identified a 35–98 nucleotide region of PVT1 that binds to the −104 to −41 bp region of the FDX1 promoter. Functionally, activation of the PVT1–FDX1 axis increases sensitivity to copper-induced proteotoxic stress in vitro and in vivo [56], as outlined in Table 3.

A complementary RNA-binding-protein-mediated mechanism has recently been identified for *SNHG7* in CRC. *SNHG7* interacts with poly(rC)-binding protein 2 (PCBP2), increases CDKN2A expression, promotes glycolytic adaptation, and suppresses cuproptosis. Functional assays and xenograft experiments support the contribution of the *SNHG7*–PCBP2–CDKN2A axis to tumour growth and cuproptosis resistance [57]. These findings demonstrate that lncRNAs can modulate cuproptosis through both epigenetic regulation of core pathway genes and post-transcriptional control of metabolic adaptation. See Table 3.

FDX1 is a central determinant of cuproptosis. It contributes to mitochondrial protein lipoylation and links copper availability to the aggregation of lipoylated TCA-cycle proteins [1]. In the context of the copper ionophore elesclomol, FDX1 also catalyses the reduction of elesclomol-bound Cu(II) to Cu(I) within mitochondria, facilitating mitochondrial copper release. However, elesclomol-mediated copper delivery is not exclusively FDX1-dependent, because partial copper release can also occur outside mitochondria through alternative mechanisms [19]. FDX1 should therefore be described as a central regulator rather than as a universal intracellular copper-reduction enzyme. See Table 3.

A subsequent study strengthened this model by showing that *SNHG7* directly interacts with PCBP2 and increases CDKN2A expression. The *SNHG7*–PCBP2–CDKN2A axis promotes glycolysis and reduces sensitivity to elesclomol–copper treatment in CRC cells [57]. A separate CRC study also reported that *SNHG7* downregulation increases *FDX1* expression and suppresses proliferation, migration, and invasion [58]. Taken together, these studies position *SNHG7* as an important candidate regulator of cuproptosis resistance, although the relationship between its FDX1-associated and CDKN2A-associated effects warrants further investigation. See Table 3.

Evidence for direct lncRNA-mediated regulation of copper transporters in tumour cuproptosis remains limited. In breast cancer (BC), transcriptomic analyses identified increased SLC31A1 expression and proposed a *LINC01614*/miR-204-5p/*SLC31A1* ceRNA axis [59]. In this model, *LINC01614* may increase *SLC31A1* expression by sequestering miR-204-5p. However, this axis should currently be described as a predicted regulatory network because direct binding assays, rescue experiments, and functional cuproptosis assays are still required. See Table 3.

These studies support the potential prognostic relevance of cuproptosis-associated transcriptional patterns. However, their interpretation requires caution. Most models were derived retrospectively from public datasets, and several lack independent external validation, prospective clinical evaluation, or experimental confirmation that the selected transcripts directly regulate cuproptosis. TIDE scores, immune-cell deconvolution analyses, and predicted drug-sensitivity estimates are computational outputs. They should not be interpreted as demonstrated responses to immune-checkpoint inhibitors, systemic treatment, or copper-ionophore therapy. See Table 3.

The current evidence supports an emerging role for lncRNAs in the regulation of cuproptosis, but the field remains substantially less developed than lncRNA-mediated ferroptosis research. The strongest mechanistic evidence currently centres on the *PVT1–FDX1* axis, which promotes cuproptotic sensitivity through epigenetic activation of *FDX1*, and the *SNHG7*–PCBP2–CDKN2A axis, which promotes glycolytic adaptation and cuproptosis resistance. See Table 3.

Future studies should prioritize direct binding assays, genetic rescue experiments, copper-ionophore response assays, and in vivo validation. Prognostic signatures remain useful for candidate discovery and patient stratification, but their translational value will depend on external validation and mechanistic confirmation. The most compelling therapeutic targets will be lncRNAs that are supported by a defined molecular mechanism and by evidence that their modulation changes sensitivity to copper-dependent cell death in vivo. See Table 3.

## 6. lncRNA-Mediated Regulation of Ferroptosis

Long non-coding RNAs regulate ferroptosis by modulating the same core modules that determine ferroptotic competence: cystine uptake, glutathione synthesis, GPX4-dependent lipid-peroxide repair, PUFA-phospholipid remodelling, iron storage or mobilization, and NRF2-centered antioxidant adaptation. However, this field is vulnerable to overinterpretation. Many proposed lncRNA-miRNA-gene axes are supported by expression correlations and gain- or loss-of-function assays, but fewer are validated at endogenous expression levels, rescued genetically, and connected to pathway-specific ferroptosis readouts. For a cancer review focused on mechanistic integration, the useful question is not whether a lncRNA is ‘ferroptosis-related’, but whether it changes the ferroptotic threshold through a defined molecular node and whether that effect is relevant to therapy response, metastatic stress, or biomarker stratification [60,61,62,63,64,65]. See Table 3.

The SLC7A11-GSH-GPX4 axis is the best-characterized lncRNA-regulated ferroptosis module. SLC7A11 maintains cystine uptake through system $x_{c}^{\text{-}}$, thereby supporting cysteine availability, glutathione synthesis, and GPX4-dependent reduction in phospholipid hydroperoxides [60,61]. lncRNAs can reinforce this axis by increasing SLC7A11 abundance, stabilizing the protein, or enhancing *GPX4* expression. *DUXAP8*, for example, suppresses ferroptosis in hepatocellular carcinoma by stabilizing SLC7A11 through enhanced palmitoylation and reduced lysosomal degradation, contributing to sorafenib resistance [62]. *OIP5-AS1* acts through a ceRNA mechanism in prostate cancer, where sequestration of miR-128-3p derepresses *SLC7A11* and limits ferroptotic injury [63]. Conversely, *SLC7A11-AS1* and *LINC00618* illustrate pro-ferroptotic lncRNA activity by reducing *SLC7A11* expression or repressing its transcription through chromatin-associated mechanisms [64,65]. See Table 3.

GPX4 is regulated by lncRNAs through ceRNA, RNA-stability, and translational mechanisms. *PVT1* can increase *GPX4* expression by sponging miR-214-3p in liver cancer cells, thereby reducing lipid peroxidation and suppressing ferroptosis [66]. *RUNX1-IT1* interacts with the m6A reader IGF2BP1 to stabilize *GPX4* mRNA and promote breast-cancer progression [67]. *lnc-HCP5* has also been linked to GPX4 regulation, and its reported peptide-coding capacity illustrates that transcripts annotated as lncRNAs may exert ferroptosis-related functions through noncanonical mechanisms [68]. These examples show that the lncRNA field should not be reduced to ceRNA models alone; RNA-binding proteins, epitranscriptomic regulation, protein turnover, and micropeptide production may all influence ferroptotic competence. See Table 3.

lncRNAs also regulate ferroptosis by controlling the membrane substrates that undergo peroxidation. ACSL4 promotes ferroptosis by activating arachidonic- and adrenic-acid-derived PUFAs for incorporation into membrane phospholipids. *ZFAS1*, *NEAT1*, and *PVT1* have been implicated in ACSL4-associated ceRNA networks, whereas *H19* and *CBSLR* can suppress ferroptosis by reducing *ACSL4* expression, activity, or stability through miRNA- and m6A/YTHDF2-associated mechanisms [69,70,71]. The key interpretation is that lncRNAs can either sensitize or protect cancer cells depending on whether they increase oxidizable PUFA-phospholipids or shift cells toward a less peroxidizable lipid state. See Table 3.

Iron-handling circuits provide another route. lncRNA *H19* has been linked to ferroptosis suppression through the miR-19b-3p/*FTH1* axis in lung cancer, suggesting that ferritin-associated iron sequestration can be regulated by lncRNA networks [72]. *CACNA1G-AS1* has been associated with IGF2BP-dependent stabilization of *FTH1* in ovarian cancer, supporting a ferritin-centred resistance mechanism [73]. More broadly, observations involving shared iron, redox, or mitochondrial regulators must be interpreted according to the strength of the evidence for genuine ferroptosis–cuproptosis interaction. Table 4 presents the framework used in this review to distinguish shared susceptibility, directional sensitization, true co-execution, and nonspecific metal-induced injury. 

lncRNA-mediated ferroptosis resistance may also support metastatic adaptation. Circulating or invading tumour cells face oxidative stress, matrix detachment, nutrient limitation, and immune pressure; suppression of lipid peroxidation can therefore facilitate survival during dissemination. *LINC02266*, for example, promotes gastric-cancer proliferation, migration, and invasion while suppressing ACSL4-dependent ferroptosis through AKT-associated signalling [74]. Prognostic lncRNA signatures may capture parts of this biology, but they should not be confused with functional mechanisms. A signature can stratify survival without proving that the included transcripts directly regulate ferroptosis or that they represent therapeutic targets. See Table 3.

The lncRNA-ferroptosis literature is relevant to the cuproptosis theme because several lncRNA-controlled nodes also regulate copper-dependent stress. Glutathione sustains GPX4 activity and buffers intracellular copper; NRF2-regulated programs can increase antioxidant capacity, metallothionein expression, detoxification enzymes, and metal sequestration; mitochondrial quality-control pathways may influence both lipid peroxidation and copper-induced proteotoxic stress. Therefore, lncRNAs that alter SLC7A11, GPX4, NRF2, ferritin, or mitochondrial metabolism may bias cancer cells toward ferroptosis resistance, cuproptosis resistance, or mixed metal-stress phenotypes. This does not mean that ferroptosis-regulating lncRNAs are automatically cuproptosis regulators. It means that they may alter shared upstream vulnerability circuits that determine how a tumour responds to metal-dependent therapeutic pressure. See Table 3.

**Figure 2:**
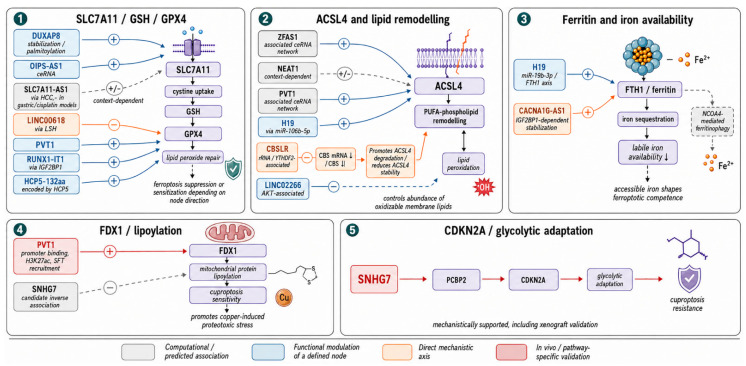
Evidence-stratified lncRNA regulatory networks controlling ferroptosis- and cuproptosis-related pathways in cancer. The schematic organizes lncRNAs according to their principal molecular targets, including the SLC7A11–GSH–GPX4 antioxidant axis, ACSL4-dependent lipid remodelling, ferritin-mediated iron sequestration, FDX1-dependent mitochondrial protein lipoylation, and SNHG7–PCBP2–CDKN2A-mediated glycolytic adaptation. Solid arrows indicate regulatory direction, dashed connectors indicate context-dependent or candidate associations, and colours represent the strength of evidence supporting each lncRNA–target axis. Note. GPAI: AI STEM Copilot (Turing Co., Ltd., Seoul, Republic of Korea) was used solely for the technical visual refinement of this author-generated figure. All scientific content, labels, and interpretations were created and verified by the authors.

For this reason, lncRNA studies should report evidence level explicitly. An evidence-based hierarchy would distinguish: (i) computational association with ferroptosis-related genes; (ii) expression change under ferroptosis-inducing conditions; (iii) functional modulation of lipid peroxidation and cell death; (iv) rescue by ferroptosis inhibitors or genetic restoration of the target node; and (v) in vivo validation with pathway-specific pharmacodynamic markers. The same logic should be applied to cuproptosis-related lncRNAs, where the relevant readouts include copper dependence, mitochondrial lipoylation, DLAT aggregation, Fe-S-protein loss, and respiratory dependence. Without these criteria, lncRNA signatures risk remaining descriptive biomarkers rather than mechanistically actionable regulators. See Table 3. The principal evidence-stratified lncRNA–target axes involved in ferroptosis and cuproptosis are summarized in Figure 2.

## 7. Mechanistic Crosstalk Between Ferroptosis and Cuproptosis in Cancer

**Figure 3:**
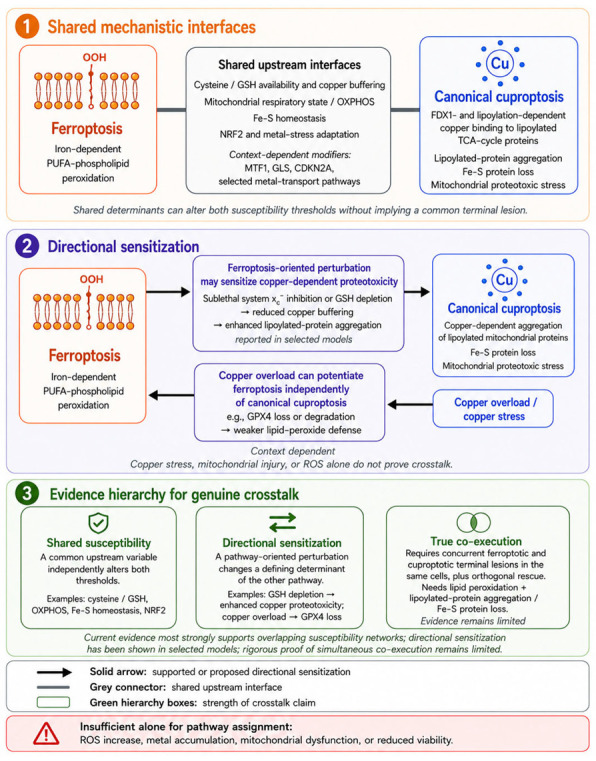
Proposed mechanistic interfaces and evidence hierarchy for ferroptosis–cuproptosis crosstalk in cancer. Ferroptosis and canonical cuproptosis retain distinct terminal lesions but may interact through shared upstream susceptibility networks and context-dependent directional sensitization. Genuine co-execution requires concurrent pathway-specific lesions and independent rescue, whereas ROS generation, metal accumulation, mitochondrial dysfunction, or reduced viability alone are insufficient to establish crosstalk. Note. GPAI: AI STEM Copilot (Turing Co., Ltd.) was used solely for the technical visual refinement of this author-generated figure. All scientific content, labels, and interpretations were created and verified by the authors.

Ferroptosis and cuproptosis retain distinct execution mechanisms, but they operate within overlapping networks of mitochondrial metabolism, thiol buffering, metal trafficking, Fe–S-cluster maintenance, and cellular stress adaptation. Their interaction is therefore most plausibly located upstream of terminal cell death rather than at a shared execution step. A perturbation that alters glutathione availability, mitochondrial respiration, or the intracellular distribution of copper and iron may shift the susceptibility threshold of both pathways without making them mechanistically identical. This distinction is essential because reactive oxygen species generation, mitochondrial dysfunction, or reduced cell viability alone cannot establish either ferroptosis or cuproptosis [1,2,3,6,45], as illustrated in Figure 3.

### 7.1. Mitochondrial Metabolism as the Principal Site of Convergence

Mitochondrial metabolism is indispensable for canonical cuproptosis. Cancer cells dependent on oxidative phosphorylation are markedly more sensitive to copper ionophores than predominantly glycolytic cells, and inhibition of mitochondrial pyruvate uptake or respiratory-chain activity reduces copper-induced death. Genome-wide screening identified *FDX1*, *LIAS*, *LIPT1*, *DLD*, *DLAT*, *PDHA1*, and *PDHB* as central determinants linking copper delivery to mitochondrial protein lipoylation. Copper binding to lipoylated DLAT and related TCA-cycle proteins promotes their oligomerization and aggregation, generating a proteotoxic lesion that is fundamentally different from nonspecific oxidative mitochondrial damage [1,6,15].

The metabolic overlap is consequently asymmetric. Increased TCA-cycle flux may expand the lipoylated mitochondrial substrate pool required for cuproptosis while also increasing oxidative pressure during cystine limitation. Conversely, strong glycolytic adaptation may reduce cuproptosis sensitivity but does not necessarily protect against direct GPX4 inhibition. Mitochondrial activity should therefore be interpreted as an obligatory determinant of canonical cuproptosis and a context-dependent modifier of ferroptosis rather than as a uniform promoter of both pathways [1,4,6,75].

### 7.2. Glutathione Couples Lipid-Peroxide Defence to Copper Buffering

Glutathione represents the most direct biochemical bridge between ferroptosis and cuproptosis. In ferroptosis, cystine uptake through system $x_{c}^{\text{-}}$− sustains glutathione synthesis and GPX4-mediated reduction in phospholipid hydroperoxides. In copper homeostasis, glutathione binds and buffers intracellular copper, limiting the bioavailable fraction capable of reaching mitochondrial targets. Glutathione depletion may therefore simultaneously weaken lipid-peroxide repair and increase the accessibility of copper to lipoylated mitochondrial proteins [1,3,6,45,76].

This interaction has been demonstrated directly in hepatocellular-carcinoma models. Erastin and sorafenib reduced cystine-dependent glutathione synthesis and enhanced the cytotoxicity of elesclomol–copper. Importantly, combined treatment increased lipoylated DLAT aggregation, providing evidence that system $x_{c}^{\text{-}}$− inhibition modified a defining cuproptosis-associated endpoint rather than merely increasing general oxidative stress. The same study linked sensitization to reduced copper–glutathione complex formation and increased FDX1 stability. These findings provide one of the strongest examples of directional crosstalk in which a ferroptosis-oriented intervention increases cuproptotic competence, as illustrated in Figure 3 [6,77,78].

### 7.3. Fe–S Clusters Link Copper Proteotoxicity to Iron Stress

Fe–S proteins form a second major interface. Cuproptosis is accompanied by FDX1-dependent loss or destabilization of Fe–S-cluster proteins, together with aggregation of lipoylated mitochondrial proteins and induction of proteotoxic stress. Fe–S proteins support electron transfer, metabolic catalysis, DNA synthesis, and iron sensing; their disruption can therefore alter mitochondrial respiration and the cellular response to iron availability. Nevertheless, lipoylated-protein aggregation remains the most clearly established proximal lesion, whereas the precise contribution of Fe–S-protein loss to cuproptotic execution is still being defined [1,6,15].

Fe–S-cluster biogenesis independently regulates ferroptosis. In lung cancer, suppression of the mitochondrial cysteine desulfurase NFS1 impairs Fe–S-cluster production and activates an iron-starvation response that increases iron uptake and sensitizes cells to ferroptosis. This establishes a mechanistic route by which disruption of mitochondrial Fe–S biology can expand the redox-active iron pool. It also raises the possibility that copper-mediated Fe–S destabilization may indirectly create ferroptosis-permissive conditions, although direct demonstration that Fe–S loss during cuproptosis is sufficient to initiate ferroptosis remains limited [3,44,79].

The Fe–S interface is particularly relevant to multifunctional copper complexes and metal-containing nanoplatforms. Such agents may simultaneously disrupt mitochondrial proteins, increase labile iron, generate lipid peroxides, and reduce antioxidant capacity. However, the concurrent detection of Fe–S-protein loss and lipid oxidation does not prove coordinated cuproptosis and ferroptosis. Each mechanism must be demonstrated independently through pathway-specific genetic perturbation, biochemical endpoints, and rescue experiments [1,6,77,80].

### 7.4. Copper–Iron Homeostasis Extends Beyond Intracellular Metal Abundance

Copper and iron homeostasis are physiologically interconnected. Copper-dependent multicopper ferroxidases, including ceruloplasmin and hephaestion, oxidize ferrous iron after ferroportin-mediated export, facilitating its loading onto transferrin and influencing tissue iron distribution. Changes in copper availability can therefore modify iron retention and the labile iron pool without directly altering ferroptosis machinery. This interaction is important in cancer because a disturbance in copper trafficking may secondarily reshape iron availability and lipid-peroxidative susceptibility [6,81,82].

### 7.5. Copper Can Directly Disable Ferroptosis Defence

Copper can promote ferroptosis through direct modification of GPX4. In pancreatic-cancer models, copper bound GPX4 at cysteine residues C107 and C148, enhanced its ubiquitination and aggregation, and facilitated TAX1BP1-mediated autophagic degradation. This mechanism reduced the capacity to detoxify phospholipid hydroperoxides and induced ferroptosis that was sensitive to copper chelation and ferroptosis inhibition. It provides direct molecular evidence that copper stress can engage ferroptosis without requiring iron–copper homeostatic crosstalk as an intermediate step [6,83].

Copper-dependent ferroptosis is nevertheless context dependent. Disulfiram–copper has induced lipid peroxidation and ferroptotic death in several experimental cancer systems, but compensatory NRF2 activation can attenuate its activity. In hepatocellular carcinoma, inhibition of the adaptive NRF2 response increased sensitivity to disulfiram–copper-induced ferroptosis. Conversely, other models indicate that copper availability may support GPX4 expression or ferroxidase activity and thereby reduce ferroptotic pressure. Copper should therefore not be classified as uniformly pro-ferroptotic; its effect depends on concentration, subcellular distribution, transporter status, antioxidant adaptation, and tumour lineage, as summarized in Figure 3 [3,6,84,85].

### 7.6. Shared Regulatory Genes Are Context Modifiers, Not Universal Dual-Pathway Switches

Several genes influence metabolic conditions relevant to both pathways. *GLS* regulates glutamine conversion to glutamate, which can support glutathione synthesis or enter the TCA cycle as α-ketoglutarate. Accordingly, glutamine metabolism may suppress ferroptosis by sustaining glutathione while promoting cystine-deprivation-induced ferroptosis through mitochondrial anaplerosis. In cuproptosis, glutathione production downstream of glutamine metabolism may buffer intracellular copper and limit mitochondrial copper availability. The effect of GLS is therefore bidirectional and determined by whether glutamate is preferentially used for antioxidant defence or mitochondrial oxidation [1,3,6,75].

*MTF1* and *CDKN2A* were identified as negative modifiers of copper-ionophore-induced death in the original genetic screen. *MTF1* supports metal buffering through metallothionein expression, whereas *CDKN2A*-associated metabolic states may favour glycolysis and reduce dependence on lipoylated mitochondrial metabolism. Both have also been linked to ferroptosis-related iron or lipid regulation in selected models. However, the evidence does not support their classification as universal executors of ferroptosis–cuproptosis crosstalk. They are better regarded as context-dependent regulators of the metabolic and metal-buffering state in which either pathway may occur [1,6,86].

The KEAP1–NRF2 pathway is similarly more established as a ferroptosis-resistance programme than as a canonical cuproptosis regulator. NRF2 increases cystine uptake, glutathione synthesis, NADPH-dependent detoxification, ferritin expression, and alternative ferroptosis defences. These same outputs can indirectly reduce bioavailable copper by increasing thiol and metal-buffering capacity, but direct evidence that NRF2 uniformly suppresses lipoylation-dependent cuproptosis remains limited. It should therefore be interpreted as an upstream redox modifier rather than a core component of the cuproptosis machinery [2,3,6,84].

### 7.7. lncRNA-Based Dual Signatures Require Functional Validation

Combined ferroptosis–cuproptosis lncRNA signatures have been described in lung adenocarcinoma, hepatocellular carcinoma, endometrial cancer, and haematological malignancies. These models may capture clinically relevant patterns of metabolism, immune infiltration, and treatment response, but they are predominantly based on co-expression, survival modelling, or computational pathway scores. Such analyses demonstrate association with gene sets from both pathways, not direct regulation of both death mechanisms [87,88,89].

A convincing dual-regulatory lncRNA should alter defining endpoints of both pathways in the same experimental system. This would require demonstrating changes in iron-dependent phospholipid peroxidation together with copper- and lipoylation-dependent mitochondrial protein aggregation, followed by independent rescue of each phenotype. Until these criteria are fulfilled, lncRNAs associated with both gene sets should be described as candidate network regulators rather than confirmed molecular mediators of ferroptosis–cuproptosis crosstalk [8,87,90].

### 7.8. Evidence Framework for Genuine Crosstalk

Current evidence supports three levels of interaction. Shared susceptibility occurs when a common variable, such as glutathione or mitochondrial activity, independently modifies both pathways. Directional sensitization occurs when manipulation of one pathway changes a defining determinant of the other, as observed when system $x_{c}^{\text{-}}$− inhibition increased copper-dependent DLAT aggregation. True co-execution requires concurrent ferroptotic lipid-peroxide injury and cuproptotic mitochondrial proteotoxicity, with each mechanism contributing independently to cell death. Most studies establish shared susceptibility, fewer demonstrate directional sensitization, and rigorous evidence for true co-execution remains limited [1,3,6,77].

Mechanistic studies should therefore combine orthogonal validation approaches. Ferroptosis requires iron-dependent phospholipid peroxidation, appropriate genetic or pharmacological rescue, and evidence that ferrostatin-1, liproxstatin-1, or iron chelation suppresses death. Cuproptosis requires copper dependence, an intact FDX1–lipoylation axis, aggregation of lipoylated mitochondrial proteins, and rescue through copper chelation or disruption of the lipoylation machinery. Demonstration of only oxidative stress, metal accumulation, or loss of viability is insufficient [1,3,5,6,45].

Taken together, the strongest evidence for ferroptosis–cuproptosis crosstalk lies in mitochondrial metabolism, glutathione buffering, Fe–S-cluster biology, and copper–iron trafficking. These pathways are neither fully independent nor interchangeable. Rather, they represent distinct terminal outcomes embedded within a connected metabolic network in which disruption of one homeostatic node may shift cancer cells toward vulnerability to the other. Preserving this distinction while defining their directional interactions is essential for interpreting experimental data and for preventing generalized metal toxicity from being misclassified as coordinated regulated cell death [1,3,6,15], as summarized in Table 4 and illustrated in Figure 3.

## 8. Therapeutic Implications

Ferroptosis and cuproptosis are therapeutically attractive because they may eliminate malignant cells that have escaped apoptosis, survived genotoxic therapy, or acquired resistance to targeted treatment. The crosstalk framework above narrows this promise by distinguishing shared susceptibility, directional sensitization, and true co-execution. Their translational value does not lie in the simple observation that lipid peroxidation or copper overload can kill cancer cells. A clinically credible strategy must define a tumour-selective dependency, engage a druggable molecular node, achieve sufficient intratumoral exposure, and include pharmacodynamic markers that prove activation of the intended regulated cell-death program rather than nonspecific oxidative injury. At present, ferroptosis has a broader experimental pharmacology and a more developed biomarker landscape, whereas cuproptosis benefits from the prior clinical development of copper ionophores such as elesclomol but still lacks prospectively validated patient-selection criteria. The next step should therefore be a shift from non-stratified use of redox-active compounds toward mechanism-guided combinations built around measurable metabolic, redox, and metal-handling vulnerabilities, as prioritized in Table 5 [3,6,91].

### 8.1. Ferroptosis as a Therapeutic Vulnerability

#### 8.1.1. Pharmacological Induction

Erastin and RSL3 remain the two pharmacological archetypes of ferroptosis induction. Erastin inhibits system $x_{c}^{\text{-}}$, the cystine-glutamate antiporter formed by SLC7A11 and SLC3A2, thereby restricting cystine import, reducing cysteine availability, and impairing glutathione synthesis. Because glutathione provides the reducing equivalents required by GPX4, this route progressively disables phospholipid-hydroperoxide repair. RSL3 acts further downstream by covalently inhibiting GPX4, allowing iron-dependent phospholipid peroxidation even before the glutathione pool is fully exhausted. This distinction is therapeutically relevant: system $x_{c}^{\text{-}}$ blockade induces a broader amino-acid and redox crisis, whereas direct GPX4 inhibition bypasses several upstream adaptations and produces a more immediate ferroptotic phenotype [3].

Neither erastin nor RSL3 should be presented as clinically mature anticancer drugs. Their main value is mechanistic: they identify tumour states in which cystine import, glutathione synthesis, or GPX4 activity has become essential for survival. Preclinical prostate-cancer models have shown that erastin and RSL3 can suppress growth, migration, and invasion and can enhance second-generation antiandrogen activity, an observation that is relevant to lineage plasticity and androgen-receptor-independent resistance. Nevertheless, the absence of overt toxicity in xenograft models does not define a human therapeutic window. For clinical translation, the field needs more stable and selective analogues, tumour-targeted formulations, enzymatic cyst(e)ine-depletion approaches, or agents that indirectly suppress *SLC7A11* expression or SLC7A11 protein stability. Sulfasalazine illustrates both the opportunity and the limitation of repurposing: it has clinical experience as a system $x_{c}^{\text{-}}$ inhibitor, but its potency, pharmacokinetics, and target selectivity make it unlikely to function as a stand-alone ferroptosis drug in unselected cancers [3,4,92].

GPX4 remains the most direct enzymatic target because it sits at the convergence of glutathione metabolism and membrane-lipid repair. Direct inhibition, proteasomal degradation, or transcriptional and translational suppression of *GPX4* can all lower the ferroptotic threshold. Compounds such as DMOCPTL in triple-negative breast cancer and rottlerin in hepatocellular carcinoma illustrate the appeal of degrading or destabilizing GPX4 and/or SLC7A11, especially when ferroptosis can be coupled to chemotherapy or targeted therapy. Upstream regulators such as STAT3 in gastric cancer and the m6A demethylase FTO in colorectal cancer provide another route, because they can maintain GPX4 and SLC7A11 while also supporting broader oncogenic programs. These strategies are attractive precisely because they do not rely on classical ferroptosis inducers alone. Their weakness is pleiotropy: reduced viability after STAT3, FTO, or proteasome-pathway modulation cannot be interpreted as ferroptosis unless lipid peroxidation and pathway-specific rescue are demonstrated, as emphasized in Table 5 [93,94,95,96].

#### 8.1.2. Biomarker-Guided Ferroptosis Therapy

A major translational lesson is that GPX4 expression alone is not sufficient to predict ferroptosis sensitivity. Ferroptosis depends on the balance between peroxidation pressure and antioxidant capacity. In addition to the system $x_{c}^{\text{-}}$-glutathione-GPX4 axis, FSP1-CoQ, GCH1-BH4, and mitochondrial DHODH-CoQ pathways can suppress lipid-radical propagation. DHODH is particularly relevant because it protects mitochondrial phospholipids and may define tumours in which GPX4 inhibition alone is insufficient or, conversely, tumours with low GPX4 expression in which DHODH blockade creates marked ferroptotic pressure. A practical biomarker model should therefore integrate *SLC7A11* and *GPX4* with ACSL4-dependent polyunsaturated phospholipid synthesis, FSP1 and DHODH activity, NRF2-KEAP1 signalling, iron handling, and tumour lipid composition [3,4].

Clinical development will also require tumour selectivity. GPX4 is not cancer-specific, and systemic inhibition could damage ferroptosis-sensitive normal tissues or impair immune populations needed for antitumor activity. More plausible strategies include tumour-restricted delivery, activatable prodrugs, antibody-drug conjugates, local administration, intermittent scheduling, or use of ferroptosis induction as a sensitizing mechanism with radiotherapy, chemotherapy, targeted therapy, or immune-checkpoint blockade. Static immunohistochemistry is unlikely to be sufficient. Patient-derived organoids, ex vivo tumour slices, and pharmacodynamic measurement of oxidized phosphatidylethanolamines, 4-hydroxynonenal, GPX4-pathway suppression, and rescue by radical-trapping antioxidants may provide a more reliable assessment of ferroptotic competence, as outlined in Table 5 [3,97].

### 8.2. Cuproptosis-Directed Therapy and Metabolic Selection

#### 8.2.1. Copper Ionophores and the Lesson from Elesclomol

Cuproptosis-directed therapy is conceptually different from simply increasing copper toxicity. Canonical cuproptosis requires mitochondrial delivery of bioavailable copper, interaction with lipoylated tricarboxylic acid-cycle proteins such as DLAT, aggregation of lipoylated proteins, destabilization of iron–sulfur cluster proteins, and mitochondrial proteotoxic stress. Elesclomol is important because it can form copper complexes and facilitate mitochondrial copper delivery, and because it entered oncology trials before cuproptosis was mechanistically defined. This trial history is informative but should be interpreted cautiously: exposure to a copper ionophore is not equivalent to verified induction of cuproptosis [1,6,98].

Earlier clinical studies of elesclomol, including melanoma trials, suggested that non-selected copper-ionophore therapy is insufficient. A post hoc signal in patients with low baseline lactate dehydrogenase is mechanistically interesting because low LDH may indicate less glycolytic dominance and greater reliance on oxidative phosphorylation, a metabolic state compatible with cuproptosis susceptibility. However, LDH also reflects tumour burden, tissue injury, and other biological processes. It should therefore be treated as an exploratory covariate rather than a stand-alone biomarker. The failure of a non-selected trial should not be read as failure of cuproptosis as a therapeutic concept; it more likely shows that metabolic and pharmacodynamic stratification were missing, as highlighted in Table 5 [6,91,98].

#### 8.2.2. Targeting the Metabolic Context of Cuproptosis

Cuproptosis sensitivity is determined by the intersection of copper handling and mitochondrial metabolic state. Tumours with high oxidative phosphorylation, intact FDX1 and lipoylation machinery, active pyruvate dehydrogenase flux, and abundant lipoylated DLAT or DLST are rational candidates for copper-ionophore therapy. This phenotype may occur in selected melanomas, leukemias, hepatocellular carcinomas, and therapy-resistant or stem-like subpopulations that have shifted toward mitochondrial metabolism. Conversely, strong glycolytic diversion, impaired mitochondrial pyruvate entry, or reduced protein lipoylation can limit the substrate pool required for canonical cuproptosis. Functional metabolic imaging, oxygen-consumption assays in patient-derived models, and proteomic or transcriptomic signatures of oxidative phosphorylation may therefore be more informative than tumour histology alone [6,91,98].

Several resistance mechanisms illustrate why single biomarkers are insufficient. In triple-negative breast cancer, AKT1-mediated phosphorylation of FDX1 has been associated with defective protein lipoylation, reduced oxidative phosphorylation, increased glycolysis, and resistance to elesclomol-induced cuproptosis; AKT inhibition restored susceptibility in experimental models. In hepatocellular carcinoma, MELK-dependent signalling can increase DLAT abundance while altering mitochondrial function and promoting elesclomol resistance, showing that total DLAT expression alone is not a reliable sensitivity marker. What matters is the functional state of the lipoylated mitochondrial proteome: localization, lipoylation, oligomerization competence, integration into active TCA-cycle complexes, and exposure to mitochondrial copper [99,100].

Metabolic combinations should therefore be designed to redirect rather than indiscriminately suppress energy metabolism. Inhibiting glycolysis or PI3K-AKT signalling may increase cuproptosis if it restores pyruvate oxidation and a lipoylation-competent mitochondrial state. By contrast, profound inhibition of both glycolysis and mitochondrial respiration may remove the machinery required for cuproptosis execution. Similarly, restricting copper export through ATP7A or ATP7B, reducing glutathione or metallothionein buffering, or using tumour-targeted copper carriers could increase mitochondrial copper availability, but systemic disruption of copper homeostasis raises hepatic, neurological, and hematologic safety concerns. Nanoparticle or activatable delivery platforms are promising only if they demonstrate controlled copper release, tumour-selective biodistribution, biodegradation, long-term safety, and cuproptosis-specific pharmacodynamics in vivo, as summarized in Table 5 [6,91,99,101,102].

### 8.3. RNA-Based Modulation of Ferroptosis and Cuproptosis Resistance

Long non-coding RNAs may be therapeutically useful because they can coordinate resistance circuits that involve metal handling, redox defence, lipid repair, or mitochondrial metabolism. Their subcellular localization should guide modality: nuclear lncRNAs may be targeted by RNase H-recruiting antisense oligonucleotides or gapmers, whereas cytoplasmic transcripts may be more suitable for siRNA-based strategies. Steric-blocking oligonucleotides can also disrupt lncRNA-protein or lncRNA-microRNA interactions without degrading the transcript. Across modalities, the main barriers remain sequence accessibility, isoform specificity, tumour delivery, endosomal escape, innate immune activation, and the limited conservation of many lncRNAs in standard animal models [8,103].

For ferroptosis, the strongest therapeutic logic is to target lncRNAs that maintain cystine import, *GPX4* activity, iron export, or ferritin-mediated iron sequestration. *SLC7A11AR* (*SLC7A11-AS1*) in lung adenocarcinoma provides a useful proof of concept because antisense oligonucleotides reduced tumour growth, lowered SLC7A11 and Ki-67, increased 4-hydroxynonenal, and enhanced the effect of imidazole ketone erastin in xenografts. Other lncRNAs, including *DUXAP8*, *PAX8-AS1*, *ITGB2-AS1*, *RP11-89*, and *CACNA1G-AS1*, illustrate how RNA circuits can influence SLC7A11 stability, *GPX4* expression, NAMPT-dependent resistance, PROM2-mediated iron export, or FTH1-associated ferritin storage. These examples should be presented as resistance-modulating strategies rather than as validated stand-alone therapeutics [62,73,104,105,106,107].

The lncRNA-cuproptosis field is less mature and requires more caution. Axes such as *LINC02362*-miR-18a-5p-*FDX1* and *PVT1-FDX1* suggest that selected transcripts may influence *FDX1* expression, cuproptosis susceptibility, and chemotherapy response. However, an oncogenic lncRNA is not necessarily a cuproptosis suppressor. A transcript may promote tumour growth while simultaneously creating a collateral sensitivity to copper-dependent death; in that setting, inhibition could unintentionally remove a therapeutic vulnerability, whereas high expression might serve as a biomarker for copper-ionophore treatment. Context dependence is also evident for lncRNAs such as *H19*, which has been linked to ferroptosis and treatment sensitivity in one setting and ATP7B-mediated copper export in another. Therefore, RNA therapeutics should be developed around a validated lncRNA-effector interaction within a defined molecular subgroup, not around the assumption that a named lncRNA has a uniform function across cancer types [8,56,108].

A useful rule for prioritization is that expression is not dependency. Candidate lncRNA targets should show reproducible dysregulation in adequately powered human cohorts, mechanistic necessity at endogenous expression levels, a predictable relationship with ferroptosis or cuproptosis pharmacodynamics, and sufficient tumour specificity to justify systemic RNA delivery. Patient-derived organoids, ex vivo tumour slices, and humanized models are particularly important for species-restricted lncRNAs. Without these criteria, lncRNA signatures risk remaining prognostic associations rather than actionable therapeutic tools, as emphasized in Table 5 [103,104].

### 8.4. Combination Strategies: Where the Integrated View Becomes Therapeutic

#### 8.4.1. Ferroptosis and Immunotherapy

The interaction between ferroptosis and antitumor immunity provides a strong rationale for combination with immune-checkpoint blockade. Activated CD8+ T cells release interferon-gamma, which can suppress *SLC3A2* and *SLC7A11* through JAK-STAT1 signalling, restrict cystine uptake, and increase ferroptotic pressure in tumour cells. Interferon-gamma can also cooperate with arachidonic-acid metabolism and ACSL4-dependent incorporation of polyunsaturated fatty acids into membrane phospholipids, increasing the oxidizable lipid substrate pool. Immune-checkpoint blockade may therefore prime malignant cells for ferroptosis, while ferroptosis induction lowers the amount of T-cell-mediated stress required to cross the lipid-peroxidation threshold [3,109].

Preclinical studies support this model when the combination is linked to a defined tumour state. In triple-negative breast cancer, a luminal androgen receptor-like subgroup characterized by fatty-acid metabolism, lipoxygenase expression, oxidative stress, and glutathione-system dependence responded more strongly to GPX4 inhibition plus PD-1 blockade than to either treatment alone. In hepatocellular carcinoma, PGAM1 inhibition induced energy stress and ferroptosis while reducing PD-L1 expression and increasing CD8+ T-cell activity. Radiotherapy may also cooperate with ferroptosis by generating lipid peroxidation and immunogenic stress. The common principle is that ferroptosis should be used to expose or amplify an immune vulnerability, not simply added as another nonspecific cytotoxic insult [110,111,112].

Ferroptosis is not uniformly immunostimulatory. Spatially restricted lipid peroxidation may promote antigen presentation and dendritic-cell activation, whereas extensive or poorly targeted ferroptosis can damage infiltrating lymphocytes, natural killer cells, or stromal compartments and may generate oxidized lipids with immunosuppressive effects. The therapeutic objective should therefore be selective ferroptosis in malignant cells within an immune-permissive microenvironment. Timing, sequence, and delivery may be as important as the drug combination itself, as reflected in the translational priorities summarized in Table 5 [3,113].

#### 8.4.2. Co-Targeting Ferroptosis and Cuproptosis

The most compelling integrated strategies are those that exploit biochemical nodes shared by ferroptosis and cuproptosis without erasing their mechanistic differences. Glutathione is the clearest example. It is required for GPX4-dependent detoxification of phospholipid hydroperoxides and also binds copper, thereby limiting the labile copper pool available for mitochondrial delivery. In hepatocellular carcinoma models, erastin or sorafenib reduced glutathione synthesis through system $x_{c}^{\text{-}}$ inhibition, increased copper availability, promoted DLAT aggregation, and sensitized cells to elesclomol-copper; glutathione replenishment attenuated this interaction. Co-induction may therefore generate reciprocal reinforcement: glutathione depletion weakens lipid-peroxide repair while simultaneously reducing copper sequestration [77].

Other shared nodes include mitochondrial metabolism, Fe-S-cluster biology, redox buffering, and metal trafficking. A glycolytic tumour may resist cuproptosis because it lacks sufficient active lipoylated TCA-cycle targets, while a ferroptosis-resistant tumour may depend on SLC7A11, GPX4, FSP1, or DHODH to suppress lipid peroxidation. Combining metabolic redirection, copper delivery, and ferroptosis sensitization could therefore be rational when each component is tied to a measurable vulnerability. By contrast, combining redox-active drugs merely because both increase ROS is mechanistically weak and may increase systemic toxicity without improving selectivity [3,6,91].

Engineered nanoplatforms that co-deliver copper, elesclomol, ferroptosis-inducing cargo, or immune-checkpoint blockade provide an experimental framework for this integrated approach. In breast- and colorectal-cancer models, combined copper and erastin delivery reduced glutathione, promoted aggregation of lipoylated mitochondrial proteins, increased lipid peroxidation, and suppressed tumour growth more effectively than single components. Other platforms attempt to increase intratumoral copper retention by inhibiting ATP7A-mediated export or by local release of copper-ionophore complexes. Although promising, the clinical translation of these platforms remains constrained by manufacturing complexity, variable intratumoral drug release, insufficient long-term toxicological data, and the difficulty of determining the relative contribution of each regulated cell-death pathway to in vivo efficacy [80,101,102].

Collectively, these approaches range from archetypal pathway inducers to indirect modulators, metabolic sensitizers, and multifunctional delivery systems. Their principal molecular actions, functional classifications, current evidence, and translational limitations are compared in Table 6.

### 8.5. Current Clinical-Trial Landscape

Clinical translation of ferroptosis-directed cancer therapy remains at an early stage. The completed phase I study NCT06048367 evaluated dose-escalated intratumoural administration of carbon nanoparticle-loaded Fe(II), CNSI-Fe(II), in 19 patients with advanced solid tumours [119]. This programme has progressed to the recruiting phase Ib/IIa study NCT07433283, which is evaluating repeated intratumoural administration of CNSI-Fe in patients with injectable advanced solid tumours [120]. A related phase II study, NCT07491783, is registered to evaluate intratumoural CNSI-Fe combined with radiotherapy in patients with head-and-neck squamous-cell carcinoma or selected soft-tissue malignancies [121]. Although these studies are explicitly framed around ferroptosis induction, their principal clinical endpoints concern safety, pharmacokinetics, tolerability, and antitumour response. Clinical activity alone would therefore not establish canonical ferroptosis without corresponding pharmacodynamic evidence of iron-dependent phospholipid peroxidation and pathway-specific engagement.

Clinical studies targeting the cystine–glutathione axis provide an additional translational route. The phase I/II SALMA study, NCT05580861, is evaluating sulfasalazine in combination with standard induction chemotherapy in patients aged 60 years or older with newly diagnosed acute myeloid leukaemia [122]. In addition to safety and clinical-response outcomes, the protocol includes measurements of glutathione, malondialdehyde, intracellular reactive oxygen species, SLC7A11 expression, NRF2-target-gene expression, and broader antioxidant-response signatures. This design may provide clinically relevant evidence of xCT inhibition and redox disruption, although these endpoints do not independently demonstrate iron-dependent phospholipid-peroxidation-driven cell death. NCT06134388 is evaluating sulfasalazine in patients with metastatic colorectal cancer. However, its registered biomarkers, including ferritin, superoxide dismutase, NF-κB, and Bax, are not specific for ferroptosis. Moreover, the registry entry was last updated in November 2023; therefore, its listed recruitment status should be interpreted cautiously [123].

The FerMDS study, NCT05924074, is an interventional diagnostic study rather than a therapeutic ferroptosis trial. It compares bone-marrow lipid peroxidation, measured using C11-BODIPY, between patients with SF3B1-mutant myelodysplastic syndromes and control participants undergoing investigation for monoclonal gammopathy of undetermined significance. The study may contribute to biomarker development and to the characterization of ferroptosis-related changes in myelodysplastic syndromes, but it does not evaluate a ferroptosis-inducing treatment [124].

Clinical investigation of copper-directed treatment remains less closely aligned with the defining criteria of canonical cuproptosis. The recruiting phase I study NCT05210374 evaluates disulfiram and copper gluconate in combination with liposomal doxorubicin in patients with treatment-refractory sarcomas. Its registered objectives include feasibility, safety, tolerability, dose determination, pharmacokinetics, and preliminary antitumour activity. The pharmacodynamic component evaluates aldehyde-dehydrogenase inhibition rather than FDX1 activity, protein lipoylation, DLAT aggregation, or Fe–S-protein destabilization. Consequently, any observed clinical activity should be described as copper-associated cytotoxicity and cannot, without additional pathway-specific evidence, be attributed to canonical cuproptosis [125].

Historical elesclomol studies provide relevant clinical context but predated the mechanistic definition of cuproptosis. The phase III SYMMETRY trial, NCT00522834, compared elesclomol plus paclitaxel with paclitaxel alone in patients with chemotherapy-naïve metastatic melanoma. The study was terminated and did not demonstrate a progression-free-survival benefit in the unselected study population, although exploratory findings suggested that baseline lactate dehydrogenase might have influenced treatment response [126]. The completed phase II study NCT00888615 evaluated elesclomol plus weekly paclitaxel in recurrent or persistent platinum-resistant ovarian, fallopian-tube, or primary peritoneal cancer. The objective response rate was 19.6%, and the investigators concluded that the regimen did not warrant further unselected clinical investigation [127]. Neither study prospectively selected patients according to oxidative-phosphorylation dependence, FDX1 activity, mitochondrial protein lipoylation, or copper-trafficking biomarkers. These trials therefore cannot retrospectively establish clinical induction of canonical cuproptosis.

Overall, the available clinical studies are more accurately described as trials of ferroptosis-oriented iron delivery, xCT inhibition, ferroptosis-related biomarker assessment, or copper-associated cytotoxicity than as definitive pathway-validated ferroptosis or cuproptosis trials. Future studies should combine biomarker-guided patient selection with paired tissue sampling and pathway-specific pharmacodynamic endpoints, including oxidized phospholipid measurements for ferroptosis and lipoylated-protein aggregation, mitochondrial metabolic dependence, and Fe–S-protein alterations for cuproptosis.

### 8.6. Tumour Contexts with Potential Therapeutic Vulnerability

The clinical prioritization of ferroptosis- and cuproptosis-directed strategies should consider both therapeutic unmet need and experimentally demonstrated pathway vulnerability. Oral squamous cell carcinoma (OSCC) is a relevant candidate because recurrence and resistance to platinum-based chemotherapy and radiotherapy remain major clinical challenges. Preclinical studies in head-and-neck cancer models have shown that pharmacological or genetic inhibition of the cystine transporter xCT/SLC7A11 induces ferroptosis and restores sensitivity to cisplatin, including in resistant xenografts. More recent analyses of OSCC further support the potential use of ferroptosis induction either as a direct antitumour strategy or as a sensitizer to conventional treatment, although clinical validation and predictive biomarkers remain unavailable [128,129]. Epithelial ovarian cancer represents another priority setting, particularly in platinum-resistant, recurrent, and stem-like disease. Strategies that increase the labile iron pool, including so-called class IV ferroptosis inducers, have produced antitumour effects in preclinical ovarian-cancer models. In addition, ovarian clear-cell carcinoma and clear-cell renal cell carcinoma display a lipid-rich, HIF-associated state that can create marked dependence on GPX4 and susceptibility to ferroptosis [130,131].

Pancreatic ductal adenocarcinoma may also be suitable for mechanism-guided ferroptosis strategies because of its pronounced dependence on extracellular cystine and antioxidant buffering. In genetically engineered mouse models, cysteine depletion induced tumour ferroptosis and suppressed pancreatic tumour progression, providing unusually strong in vivo evidence for exploiting this metabolic dependency [132]. Therapy-resistant melanoma represents a different but complementary setting. Melanoma differentiation and dedifferentiation states are associated with distinct ferroptosis sensitivities, and the cell-state transitions accompanying resistance to targeted therapy or immune pressure may increase vulnerability to iron-dependent oxidative death [133]. More broadly, drug-tolerant persister cells derived from melanoma, breast, lung, and ovarian cancer models acquire GPX4 dependence; GPX4 disruption selectively eliminated these residual cells and delayed tumour relapse in mice [134]. These observations suggest that ferroptosis-directed interventions may be particularly valuable as treatment-sensitizing or relapse-preventing strategies rather than as unselected monotherapies.

Histology-specific evidence for canonical cuproptosis remains less developed. A recent mechanistic study identified KRAS-mutant lung adenocarcinoma as a potentially relevant context: these tumours relied on ATP7A-mediated copper export, whereas elesclomol promoted OSTM1-dependent ATP7A degradation, intracellular copper accumulation, and cuproptotic tumour suppression in cancer cells, organoids, and genetically engineered mouse models [28]. Historical clinical experience with elesclomol nevertheless illustrates the limitations of tumour-type selection without metabolic stratification. In an unselected phase III melanoma population, elesclomol plus paclitaxel did not significantly improve progression-free survival, although a prospectively defined subgroup with normal baseline lactate dehydrogenase showed a favourable signal [135]. Similarly, a phase II study in platinum-resistant ovarian, fallopian-tube, or primary peritoneal cancer reported acceptable tolerability but insufficient overall activity to support further unstratified investigation [136]. Because these trials predated the definition of cuproptosis, they cannot establish cuproptotic pathway engagement. Collectively, the available evidence indicates that tumour histology should serve only as an initial enrichment criterion; selection should ultimately incorporate oxidative-phosphorylation dependence, functional protein lipoylation, mitochondrial copper delivery, ferroptosis-defence dependency, and pathway-specific pharmacodynamic confirmation [1,3,6,28,135,136].

### 8.7. Translational Priorities

A clinically credible development program should prioritize interpretable mechanisms and available pharmacodynamic assays. Ferroptosis-immunotherapy combinations could enrich for tumours with ACSL4-dependent lipid remodelling, SLC7A11 or GPX4 dependence, interferon-gamma responsiveness, and an existing but functionally restrained CD8+ T-cell infiltrate. Cuproptosis trials should enrich for oxidative phosphorylation dependence, intact FDX1 and lipoylation machinery, low glycolytic dominance, and measurable mitochondrial copper accumulation. Paired biopsies should assess oxidized phospholipids and GPX4-pathway suppression for ferroptosis, and DLAT aggregation, Fe-S-protein loss, lipoylation status, and labile mitochondrial copper for cuproptosis. Circulating LDH may be incorporated as an exploratory covariate in copper-ionophore trials, but not as the sole inclusion biomarker [3,6,91].

**Figure 4:**
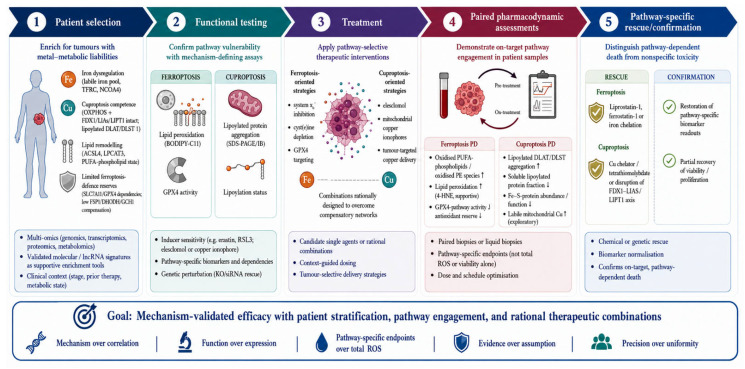
Proposed translational roadmap for mechanism-guided targeting of ferroptosis and cuproptosis in cancer. Candidate patients are enriched using integrated measures of metal handling, lipid remodelling, antioxidant-defence dependency, mitochondrial metabolism and protein lipoylation, followed by functional validation in patient-derived models. Pathway-selective or rationally combined interventions should be accompanied by paired pharmacodynamic sampling. Ferroptosis engagement should be supported by iron-dependent phospholipid peroxidation and rescue by radical-trapping antioxidants or iron chelation, whereas canonical cuproptosis should be supported by copper-dependent aggregation of lipoylated mitochondrial proteins, Fe–S-protein loss, and rescue by copper chelation or disruption of the FDX1–LIAS/LIPT1 axis. Expression signatures, total ROS, metal accumulation or reduced viability alone are insufficient for pathway assignment. Rescue experiments refer to matched ex vivo or preclinical systems rather than direct rescue treatment in patients. Note. GPAI: AI STEM Copilot (Turing Co., Ltd.) was used solely for the technical visual refinement of this author-generated figure. All scientific content, labels, and interpretations were created and verified by the authors.

Ultimately, the clinical value of ferroptosis and cuproptosis will depend less on whether these pathways can be induced and more on whether they can be induced selectively in the correct patient at the correct treatment stage. Ferroptosis is currently closest to translation as a sensitizing mechanism alongside radiotherapy, chemotherapy, targeted therapy, or immune-checkpoint blockade, rather than through unrestricted systemic GPX4 inhibition. Cuproptosis is most plausibly developed through biomarker-selected copper-ionophore therapy combined with metabolic manipulation or tumour-restricted copper delivery. RNA therapeutics may refine both approaches by disabling tumour-specific resistance circuits, but they remain at an early preclinical stage. The field should now move from proof-of-toxicity to proof-of-mechanism: functional metabolic profiling, pathway-specific pharmacodynamic markers, and patient-derived models should precede large efficacy trials if metal-dependent cell death is to become a reproducible anticancer strategy rather than a compelling preclinical concept, as summarized in Table 5 and illustrated in Figure 4 [3,6,8].

## 9. Challenges and Future Directions

Several challenges still limit the translation of ferroptosis and cuproptosis into precision oncology. First, pathway assignment remains inconsistent. Cuproptosis still lacks standardized assays able to distinguish canonical mitochondrial proteotoxicity from nonspecific copper injury, while ferroptosis studies still sometimes rely on general ROS or viability readouts instead of lipid-peroxidation and rescue criteria. Second, tumour heterogeneity may obscure vulnerable cellular niches in bulk datasets. Subclones, immune cells, stromal compartments, and metabolic microenvironments may differ substantially in iron handling, copper trafficking, mitochondrial state, and antioxidant buffering.

Future studies should therefore combine pathway-specific pharmacodynamics with single-cell, spatial, lipidomic, metallomic, and patient-derived functional models. Therapeutic specificity is equally important because iron metabolism, copper homeostasis, mitochondrial respiration, and lipid redox balance are essential for normal tissues. Tumour-selective delivery, intermittent or local exposure, rational combinations, and biomarker-guided patient selection will be required before systemic ferroptosis or cuproptosis induction can be clinically credible.

## 10. Conclusions

Ferroptosis and cuproptosis are best understood as distinct but connected metabolic vulnerabilities in cancer. Ferroptosis reflects failure of iron-dependent phospholipid-peroxide control, whereas cuproptosis reflects copper-triggered disruption of a lipoylated mitochondrial proteome. Their translational value will depend on separating pathway dependency from expression association, validating genetic and lncRNA regulators through functional rescue and pharmacodynamic endpoints, and selecting tumours by integrated measures of iron handling, copper trafficking, lipid remodelling, antioxidant capacity, mitochondrial metabolism, and protein lipoylation.

The field should now move from descriptive signatures and proof-of-toxicity models toward mechanism-guided patient stratification. Tumours should be classified not simply as ferroptosis-related or cuproptosis-related, but as ferroptosis-prone, cuproptosis-prone, co-vulnerable, or resistant according to functional evidence. Preserving the distinction between regulated cell death and nonspecific metal-induced toxicity is essential if metal-dependent vulnerabilities are to become reproducible anticancer strategies rather than compelling preclinical concepts.

## Figures and Tables

**Table 1:** Selected genetic determinants and modifiers of canonical cuproptosis.

Functional Category	Gene	Encoded Protein or Function	Role in Cuproptosis
Central determinant	*FDX1*	Mitochondrial ferredoxin 1	Promotes LIAS-dependent lipoylation and facilitates reduction of ionophore-bound Cu(II) to Cu(I)
Lipoylation machinery	*LIAS*	Lipoyl synthase	Required for mitochondrial protein lipoylation
Lipoylation machinery	*LIPT1*	Lipoyltransferase 1	Supports lipoyl-group transfer to mitochondrial proteins
Lipoylated effector	*DLAT*	PDH E2 component	Best-characterized aggregation-prone lipoylated effector
Lipoylation-dependent proteins	*DLST*, *DBT*, *GCSH*	Mitochondrial metabolic proteins	Contribute to the broader lipoylation-dependent metabolic context
Metabolic integration	*DLD*	Dihydrolipoamide dehydrogenase	Shared E3 component; proposed alternative upstream electron donor for FDX1
Metabolic integration	*PDHA1*, *PDHB*	PDH E1 components	Link pyruvate metabolism to cuproptotic competence
Negative regulation	*MTF1*, *GLS*, *CDKN2A*	Stress-response and metabolic regulators	Associated with resistance to cuproptotic stress
Copper uptake	*SLC31A1*/CTR1	High-affinity copper importer	Increases intracellular copper availability
Copper trafficking/export	*ATP7A*, *ATP7B*	Copper-transporting ATPases	Support intracellular trafficking and copper export

**Table 2:** Selected genetic modules and evidence-based interpretation.

Module	Representative Genes	Primary Role	Recommended Interpretation
Cuproptosis core lipoylation machinery	*FDX1*, *LIAS*, *LIPT1*, *DLD*, *DLAT*, *DLST*	Controls the lipoylated mitochondrial substrate pool and copper-triggered aggregation	High mechanistic relevance; functional lipoylation and DLAT aggregation matter more than mRNA alone
Copper uptake/export	*SLC31A1*/CTR1, *ATP7A*, *ATP7B*	Controls bioavailable copper and mitochondrial copper pressure	Transporter expression requires functional copper and pharmacodynamic validation
Iron uptake and mobilization	*TFRC*, *STEAP3*, *SLC11A2*, *NCOA4*	Expands labile iron pool	Core ferroptosis module, but insufficient without lipid substrates and weak antioxidant defence
Iron storage/export	*FTH1*, *FTL*, *SLC40A1*	Sequesters or exports iron	Resistance module; interpret with NCOA4 and TFRC
PUFA activation/incorporation	*ACSL4*, *LPCAT3*, *ELOVL5*, *FADS1/2*	Generates peroxidizable membrane phospholipids	Strong determinant; context varies by lineage, inducer, and lipid supply
MUFA-mediated resistance	*SCD1*, *ACSL3*	Reduces relative PUFA-phospholipid burden	Useful membrane-state marker
Canonical antioxidant defence	*SLC7A11*, *SLC3A2*, *GCLC*, *GCLM*, *GSS*, *GSR*, *GPX4*	Maintains cystine-GSH-GPX4 lipid-peroxide repair	Central therapeutic module; cystine deprivation and GPX4 inhibition are mechanistically distinct
Parallel antioxidant defence	*AIFM2*/*FSP1*, *DHODH*, *GCH1*, *DHFR*	Provides GPX4-independent radical trapping	Explains resistance to single-node ferroptosis induction
Oncogenic/tumour-suppressor context	*TP53*, *KEAP1*, *NFE2L2*, *MYC*, *BAP1*	Reprograms iron, lipid, antioxidant, and metabolic modules	Do not use as single-gene labels; interpret through downstream network

**Table 3:** Representative lncRNA evidence levels in metal-dependent cell-death biology.

Evidence Level	What It Supports	Representative Examples	Minimum Requirement Before Therapeutic Interpretation
Computational association	Candidate link to pathway-related genes or prognosis	Cuproptosis-related signatures in BLCA, ccRCC, HCC, LUSC, NSCLC	External validation and functional testing
Functional modulation of a defined node	Regulatory role	*DUXAP8*-SLC7A11; *OIP5-AS1*-SLC7A11; *PVT1*-GPX4; *RUNX1-IT1*-GPX4	Pathway-specific biochemical endpoint plus target rescue
Direct mechanistic axis	Higher-confidence regulator	*PVT1*-FDX1; *SNHG7*-PCBP2-CDKN2A; *LINC00618*-LSH-SLC7A11	Direct binding, target rescue, and pathway-specific pharmacodynamics
In vivo/pathway-specific validation	Candidate therapeutic or stratification tool	*SLC7A11-AS1* ASO; selected validated resistance axes	Tumour-relevant delivery, safety, and biomarker-linked response

**Table 4:** Evidence framework for interpreting ferroptosis–cuproptosis crosstalk.

Level	Definition	Example Logic	Evidence Required
Shared susceptibility	One upstream variable independently changes both thresholds	Glutathione depletion, mitochondrial state, NRF2 activity	Separate pathway readouts showing altered sensitivity
Directional sensitization	Manipulating one pathway changes a defining determinant of the other	System $x_{c}^{\text{-}}$ inhibition increases DLAT aggregation; copper promotes GPX4 degradation	Target-node change plus pathway-specific rescue
True co-execution	Both terminal lesions contribute independently to death	Lipid peroxidation and lipoylated-protein aggregation in same system	Independent and combined rescue of ferroptosis and cuproptosis endpoints
Misclassification	Generic metal toxicity interpreted as regulated death	ROS increase, metal accumulation, mitochondrial dysfunction, reduced viability	Insufficient without pathway-defining biochemical markers

**Table 5:** Translational prioritization of therapeutic strategies targeting ferroptosis and cuproptosis.

Strategy	Main Dependency	Candidate Biomarkers	Main Limitation
System $x_{c}^{\text{-}}$ inhibition/cysteine depletion	Cystine import and glutathione synthesis	*SLC7A11* dependence, low cysteine reserve, *ACSL4*-high lipid state, GPX4 reliance	Limited selectivity and compensatory antioxidant systems
Direct or indirect GPX4 suppression	Phospholipid-hydroperoxide repair	GPX4 function, oxidized phospholipids, FSP1/DHODH/GCH1 status	Normal-tissue and immune-cell toxicity if systemic
Ferroptosis plus immunotherapy or radiotherapy	Therapy-induced lipid-peroxidative stress	Interferon-gamma response, CD8+ infiltrate, *SLC7A11*/GPX4 dependence	Timing, delivery, and immune-context dependence
Elesclomol/copper-ionophore therapy	Mitochondrial copper delivery and lipoylated-protein aggregation	OXPHOS dependence, *FDX1*, lipoylation status, DLAT aggregation, mitochondrial copper	No prospectively validated selection biomarker
Metabolic sensitization to cuproptosis	Pyruvate/TCA flux and active lipoylated proteome	Oxygen consumption, PDH/DLAT/DLST lipoylation, low glycolytic dominance	Over-suppression of metabolism may block execution
RNA-based resistance modulation	lncRNA-controlled *SLC7A11*, *GPX4*, *FDX1*, *CDKN2A*, ferritin, or transporter axes	Validated lncRNA-effector dependency and pathway-specific pharmacodynamics	Delivery, isoform specificity, and expression-dependency mismatch
Co-induction/nanoplatform delivery	Shared glutathione, metal-buffering, mitochondrial, or lipid-peroxidation nodes	Glutathione depletion, lipid peroxidation, DLAT aggregation, copper retention	Manufacturing, toxicology, biodistribution, and pathway attribution

**Table 6:** Representative pharmacological inducers and sensitizing strategies targeting ferroptosis and cuproptosis in cancer.

Pathway	Agent or Strategy	Principal Molecular Action	Functional Classification	Current Evidence and Main Translational Limitation
**Ferroptosis**	Erastin	Inhibits system $x_{c}^{\text{-}}$, restricts cystine uptake, lowers glutathione synthesis, and indirectly impairs GPX4-dependent phospholipid-hydroperoxide repair.	Archetypal indirect ferroptosis inducer	Widely used in mechanistic and preclinical models; limited stability, pharmacokinetics, and clinical applicability.
	Imidazole ketone erastin (IKE)	A more stable erastin analogue that inhibits system $x_{c}^{\text{-}}$ and promotes cystine deprivation and glutathione depletion.	Optimized indirect ferroptosis inducer	Preclinical agent with improved properties relative to erastin; no established human therapeutic window.
	Sulfasalazine	Inhibits xCT/SLC7A11-mediated cystine transport and weakens glutathione-dependent antioxidant defence.	Repurposed system $x_{c}^{\text{-}}$ inhibitor and sensitizer	Clinically available for inflammatory disease, but limited potency, selectivity, and tumour exposure constrain oncology use.
	RSL3	Covalently inhibits GPX4, directly preventing detoxification of phospholipid hydroperoxides.	Archetypal direct GPX4 inhibitor	Strong mechanistic tool in preclinical studies; not clinically mature and lacks an established systemic safety profile.
	DMOCPTL	Binds GPX4 and promotes its ubiquitination and degradation.	Indirect GPX4-targeting inducer	Preclinical evidence, including triple-negative breast-cancer models; pleiotropic effects and clinical selectivity remain unresolved.
	Rottlerin	Promotes proteasome-dependent loss of SLC7A11 and GPX4, weakening both cystine uptake and lipid-peroxide repair.	Dual-node indirect inducer and sensitizer	Preclinical evidence in hepatocellular carcinoma; pleiotropy and incomplete definition of the degradation machinery limit interpretation.
	Sorafenib	Can inhibit system $x_{c}^{\text{-}}$ and reduce glutathione-dependent defence in selected tumour contexts.	Context-dependent inducer or sensitizer	Clinically approved multikinase inhibitor, but ferroptosis is not universal and may coexist with other mechanisms of cytotoxicity.
**Cuproptosis**	Elesclomol–copper	Facilitates intracellular and mitochondrial copper delivery, enabling copper engagement and aggregation of lipoylated TCA-cycle proteins.	Prototypical copper-ionophore strategy	Strong mechanistic and preclinical rationale with prior clinical experience; requires biomarker-guided selection and pathway-specific pharmacodynamic confirmation.
	AKT inhibition plus elesclomol–copper	Restores FDX1-associated mitochondrial metabolism and protein lipoylation, thereby increasing susceptibility to copper-ionophore treatment.	Metabolic sensitization strategy	Preclinical evidence in selected models; requires broader validation and confirmation of dependence on the proposed AKT1–FDX1 mechanism.
**Ferroptosis–cuproptosis crosstalk**	Erastin or sorafenib plus elesclomol–copper	Glutathione depletion weakens lipid-peroxide repair, reduces copper sequestration, increases copper availability, and enhances lipoylated-protein aggregation.	Directional sensitization and rational co-targeting	Promising preclinical evidence; the contribution of each death pathway must be demonstrated with independent endpoints and rescue experiments.
	Tumour-targeted copper and ferroptosis-inducing nanoplatforms	Co-deliver copper, copper ionophores, or ferroptosis-inducing cargo and may also reduce copper export or enhance intratumoural metal retention.	Multifunctional delivery and co-induction strategy	Preclinical only; manufacturing complexity, biodistribution, long-term toxicology, and pathway attribution remain major barriers.

## Data Availability

No new data were created or analyzed in this study. Data sharing is not applicable to this article.

## Abbreviations

The following abbreviations are used in this manuscript:

ACSL4	acyl-CoA synthetase long-chain family member 4
AI	artificial intelligence
ASO	antisense oligonucleotide
ATP	adenosine triphosphate
BC	breast cancer
BH4	tetrahydrobiopterin
BLCA	bladder urothelial carcinoma
ccRCC	clear-cell renal cell carcinoma
CD8+ T cell	cluster of differentiation 8-positive T cell
ceRNA	competing endogenous RNA
CoA	coenzyme A
CoQ	coenzyme Q
CRC	colorectal cancer
CRISPR	clustered regularly interspaced short palindromic repeats
CTR1	copper transporter 1
DHFR	dihydrofolate reductase
DHODH	dihydroorotate dehydrogenase
DLAT	dihydrolipoamide S-acetyltransferase
DLD	dihydrolipoamide dehydrogenase
DLST	dihydrolipoamide S-succinyltransferase
DMT1	divalent metal transporter 1
DNA	deoxyribonucleic acid
FDX1	ferredoxin 1
Fe–S	iron–sulfur
FSP1	ferroptosis suppressor protein 1
GCH1	GTP cyclohydrolase 1
GPAI	generative artificial intelligence
GPX4	glutathione peroxidase 4
GSH	reduced glutathione
HCC	hepatocellular carcinoma
LDH	lactate dehydrogenase
LIAS	lipoyl synthase
LIPT1	lipoyltransferase 1
lncRNA	long non-coding RNA
LPCAT3	lysophosphatidylcholine acyltransferase 3
LUSC	lung squamous cell carcinoma
m6A	N6-methyladenosine
miRNA	microRNA
mRNA	messenger RNA
MUFA	monounsaturated fatty acid
NAD(P)H	reduced nicotinamide adenine dinucleotide phosphate and/or reduced nicotinamide adenine dinucleotide
NADPH	reduced nicotinamide adenine dinucleotide phosphate
NRF2	nuclear factor erythroid 2-related factor 2
NSCLC	non-small-cell lung cancer
OSCC	oral squamous cell carcinoma
OXPHOS	oxidative phosphorylation
PCBP2	poly(rC)-binding protein 2
PD-1	programmed cell death protein 1
PD-L1	programmed death-ligand 1
PDH	pyruvate dehydrogenase
PUFA	polyunsaturated fatty acid
RNA	ribonucleic acid
ROS	reactive oxygen species
siRNA	small interfering RNA
TCA	tricarboxylic acid
TFRC	transferrin receptor 1
TIDE	Tumour Immune Dysfunction and Exclusion
TNBC	triple-negative breast cancer
